# Attention-deficit/hyperactive disorder updates

**DOI:** 10.3389/fnmol.2022.925049

**Published:** 2022-09-21

**Authors:** Miriam Kessi, Haolin Duan, Juan Xiong, Baiyu Chen, Fang He, Lifen Yang, Yanli Ma, Olumuyiwa A. Bamgbade, Jing Peng, Fei Yin

**Affiliations:** ^1^Department of Pediatrics, Xiangya Hospital, Central South University, Changsha, China; ^2^Hunan Intellectual and Developmental Disabilities Research Center, Changsha, China; ^3^Department of Neurology, Children’s Hospital Affiliated to Zhengzhou University, Henan Children’s Hospital, Zhengzhou Children’s Hospital, Zhengzhou, China; ^4^Department of Anesthesiology and Pharmacology, University of British Columbia, Vancouver, BC, Canada

**Keywords:** attention-deficit/hyperactive disorder, pathogenic pathways, environmental factors, genes, copy number variations, microRNAs (miRNAs)

## Abstract

**Background:**

Attention-deficit/hyperactive disorder (ADHD) is a neurodevelopmental disorder that commonly occurs in children with a prevalence ranging from 3.4 to 7.2%. It profoundly affects academic achievement, well-being, and social interactions. As a result, this disorder is of high cost to both individuals and society. Despite the availability of knowledge regarding the mechanisms of ADHD, the pathogenesis is not clear, hence, the existence of many challenges especially in making correct early diagnosis and provision of accurate management.

**Objectives:**

We aimed to review the pathogenic pathways of ADHD in children. The major focus was to provide an update on the reported etiologies in humans, animal models, modulators, therapies, mechanisms, epigenetic changes, and the interaction between genetic and environmental factors.

**Methods:**

References for this review were identified through a systematic search in PubMed by using special keywords for all years until January 2022.

**Results:**

Several genes have been reported to associate with ADHD: *DRD1*, *DRD2*, *DRD4*, *DAT1*, *TPH2*, *HTR1A*, *HTR1B*, *SLC6A4*, *HTR2A*, *DBH*, *NET1*, *ADRA2A*, *ADRA2C*, *CHRNA4*, *CHRNA7*, *GAD1*, *GRM1*, *GRM5*, *GRM7*, *GRM8*, *TARBP1*, *ADGRL3*, *FGF1*, *MAOA*, *BDNF*, *SNAP25*, *STX1A*, *ATXN7*, and *SORCS2*. Some of these genes have evidence both from human beings and animal models, while others have evidence in either humans or animal models only. Notably, most of these animal models are knockout and do not generate the genetic alteration of the patients. Besides, some of the gene polymorphisms reported differ according to the ethnic groups. The majority of the available animal models are related to the dopaminergic pathway. Epigenetic changes including SUMOylation, methylation, and acetylation have been reported in genes related to the dopaminergic pathway.

**Conclusion:**

The dopaminergic pathway remains to be crucial in the pathogenesis of ADHD. It can be affected by environmental factors and other pathways. Nevertheless, it is still unclear how environmental factors relate to all neurotransmitter pathways; thus, more studies are needed. Although several genes have been related to ADHD, there are few animal model studies on the majority of the genes, and they do not generate the genetic alteration of the patients. More animal models and epigenetic studies are required.

## Introduction

Attention-deficit/hyperactive disorder (ADHD) is a neurodevelopmental disorder that is characterized by hyperactivity, inattention, impulsivity, and problems in social interaction and academic performance [Diagnostic and Statistical Manual of Mental Disorders, 5th edition (DSM-5)]. The predominantly hyperactive/impulsive, predominantly inattentive, and combined types are the major three forms that exist (DSM-5). It most commonly occurs in children with prevalence rates ranging from 3.4 to 7.2% (Polanczyk et al., 2015; Thomas et al., 2015). It affects children aged 6 to 17 years (Berger, 2011); however, it can persist into adulthood in about 60 to 80% (Childress and Berry, 2012). Males are more affected compared to females (Childress and Berry, 2012). This disorder profoundly affects the academic achievement, well-being, and social interactions of children (Klein et al., 2012; Groman and Barzman, 2014), as a result, it brings high costs for both individuals and society (Pelham et al., 2007).

The phenotype is complex and heterogeneous, presenting with variable clinical features, developmental course, and outcome. The diagnosis of ADHD is typically determined in the school-age years; however, it can occur from early childhood. It is very difficult to diagnose young children due to their inability to cooperate well, and this happens because diagnosis relies much on interviews and observations according to the rating scale. There is no available standardized protocol for the auxiliary examinations which can assist in diagnosis despite the presence of many studies. Moreover, ADHD is frequently comorbid with other conditions in about 75% of the cases (Pliszka, 1998; Fayyad et al., 2007), including anxiety disorders, oppositional defiant disorder, mood disorders, substance use disorders, and conduct disorders (Rizzo et al., 2010; Nogueira et al., 2014; Tokko et al., 2022). All the aforementioned issues make this disorder more complex (Dias et al., 2013).

Although the etiology and pathophysiology remain unclear, there is some evidence supporting it to occur due to an interaction between genetic and environmental factors (Sun et al., 2018). Neurotransmitters including dopamine and norepinephrine acting in conjunction with each other *via* multiple receptors influence the functioning of the prefrontal cortex, cerebellum, and caudate, which are responsible for the regulation of attention, thoughts, emotions, behavior, and actions (Pliszka, 2005; Arnsten and Pliszka, 2011). These areas have also emerged as primary areas showing deficits in ADHD (Arnsten and Pliszka, 2011). Polymorphisms of the genes that encode dopamine receptor D4 (DRD4), dopamine transporter 1 (DAT-1), and 5-hydroxytryptamine receptor 1 (HTR1) have been reported to cause dysfunction of the neurotransmission (Gizer et al., 2009; Akutagava-Martins et al., 2013; Bonvicini et al., 2018). Microelements deficiencies including zinc, magnesium, and iron can affect the production of neurotransmitters (Villagomez and Ramtekkar, 2014; Hariri and Azadbakht, 2015). Despite multiple studies on ADHD, the pathogenesis is not clear, hence, the existence of many challenges especially in making an early and correct diagnosis, and provision of accurate management.

Consequently, we aimed to review the pathogenic pathways of ADHD in children. The major focus was to provide an update of the reported etiologies in humans, animal models, modulators, mechanisms, therapies (pharmacological and dietary), and the interaction between genetic and environmental factors. This update will deepen the understanding of the pathogenesis, the interaction between genetic and environmental factors, and possibly provide molecular targets for the development of accurate therapies.

## Methods

References for this review were identified through a systematic search in PubMed. Keywords used were the combination of Attention-Deficit/Hyperactivity Disorder or ADHD and gene or copy number variations or microRNAs or environmental factors or social factors or diet or epigenetics or neuroimaging or animal model or modulators, or pharmacology or mechanisms or pathways for all years until January 2022. This review included only papers published in English regarding ADHD, animal models, gene mutations, copy number variations, micro-RNAs, neuro-imaging changes, environmental factors, epigenetic changes, pathways, dietary, and pharmacological treatments. It excluded abstracts, patents, book chapters, and conference papers. The final reference list was generated based on the originality and relevance to the broad scope of this review.

## Results

Several genes have been related to ADHD in human beings from different parts of the world as shown in Supplementary Table 1. There are some microRNAs reported to relate to ADHD (Supplementary Table 2). Environmental and social biomarkers reported to relate to ADHD can be found in Supplementary Table 3. Table 1 provides a summary of different neurotransmitters and neuroreceptors and the roles they play in the pathophysiology and pathogenesis of ADHD, as well as their interactions with environmental/social factors and their respective pharmacology. Genes reported to associate with ADHD in animal models and modulators have been summarized in Table 2. Detailed results and discussion of the common pathogenic pathways and the interaction between genetic and environmental factors can be found in the following sections.

**TABLE 1:** A summary of different neurotransmitters and neuroreceptors and the roles they play in the pathophysiology and pathogenesis of ADHD.

Neurotransmitters	Neuronal interactions	Role of the neurotransmitter	Neuro-receptors	Neuronal location	Distribution	Role (s) in the pathophysiology and pathogenesis of ADHD	Microelement/ social/environmental factor interaction	Pharmacology
Dopamine	GABAergic, serotonergic, adrenergic, glutamatergic, and cholinergic neurons (Robertson and Staines, 1994; Hersch et al., 1995; Centonze et al., 2002, 2003; Rivera et al., 2002, 2003; Berlanga et al., 2005; Mizuno et al., 2007; Xi and Gardner, 2007; Oades, 2008; Saunders et al., 2015).	Regulate locomotion, reward, reinforcement, memory, and learning (Snyder, 2011).	DRD1	Postsynaptic membrane (Navandar et al., 2021).	Prefrontal cortex (Navandar et al., 2021), striatum and substantia nigra (Gagnon et al., 2017).	Postsynaptic neurotransmission of dopamine (Navandar et al., 2021).	Iron (Youdim et al., 1983; Biederman, 2005; Burhans et al., 2005; Daubner et al., 2011; Hyacinthe et al., 2015; Ranganath and Jacob, 2016). Zinc (Akhondzadeh et al., 2004; Arnold et al., 2011; Tippairote et al., 2017). Ethanol (Gibson et al., 2000). Nicotine (Paz et al., 2007). Anoxia (Miguel et al., 2018). Inflammatory cytokines (Myint and Kim, 2014)	Methylphenidate (Russell, 2011). Fluoxetine (Gainetdinov et al., 1999; Barr et al., 2004). Amphetamines (Faraone, 2018).
			DRD4	Postsynaptic membrane (Noaín et al., 2006; Leung et al., 2017).	Prefrontal cortex (Noaín et al., 2006; Leung et al., 2017).	Responsible for postsynaptic transmission of dopamine (Noaín et al., 2006; Leung et al., 2017).		
			DRD2	Both presynaptic and postsynaptic membranes (Usiello et al., 2000).	Prefrontal cortex (Usiello et al., 2000).	Postsynaptic neurotransmission of norepinephrine (Robbins et al., 1988).		
			DAT1	Presynaptic membrane (Diamond, 2007).	Striatum and prefrontal cortex (Diamond, 2007).	Mediates the reuptake of dopamine from the synapse and primary regulator of dopaminergic neurotransmission (Ernst et al., 1999).		
Serotonin	GABAergic dopaminergic, glutamatergic, and acetylcholine neurons (Lucki, 1998; Lladó-Pelfort et al., 2012; Zuo et al., 2015)	Regulate cognition, behavior, and immunity (Puig and Gulledge, 2011).	TPH2	Presynaptic membrane (Gutknecht et al., 2009, 2012)	Frontal cortex (Gutknecht et al., 2009, 2012)	Rate-limiting enzyme in the synthesis of serotonin from tryptophan (Pratelli and Pasqualetti, 2019).	Anoxia (Miguel et al., 2018). Inflammatory cytokines (Myint and Kim, 2014).	Methylphenidate (Millan et al., 2000; Oades, 2008). Fluoxetine (Gainetdinov et al., 1999; Barr et al., 2004).
			HTR1A	Postsynaptic membrane (Brown et al., 2005).	Limbic areas, hypothalamus, and cortex (Brown et al., 2005).	Regulate dopamine and serotonin levels in the brain (Lucki, 1998; Zuo et al., 2015).		
			HTR2A	Postsynaptic and glial cell membrane (Hou et al., 2018)	Neocortex, caudate nucleus, nucleus accumbens, and hippocampus (Hou et al., 2018)	It facilitates the reuptake of serotonin from the synapse to the glial cell and postsynaptic membrane. Activation of a subpopulation of GABAergic neurons (Serretti et al., 2007).		
			HTR1B	Presynaptic membrane (Hou et al., 2018)	Striatum, basal ganglia, and hippocampus (Hou et al., 2018)	It controls the release of dopamine, serotonin, and acetylcholine in the brain (Lucki, 1998; Zuo et al., 2015).		
			SLC6A4	Presynaptic membrane (Bengel et al., 1997)	Frontal cortex (Bengel et al., 1997)	Transports the neurotransmitter serotonin from synaptic spaces into presynaptic neurons (Bengel et al., 1997).		
Norepinephrine	Dopaminergic neurons (Lei, 2014).	Crucial for cognition, memory, regulation of stress, and uptake of lactate to the neurons (Borodovitsyna et al., 2017; Gonzalez-Lopez and Vrana, 2020).	DBH	Synaptic vesicles and post-ganglionic sympathetic fibers (Gonzalez-Lopez and Vrana, 2020).	Brain cortex (Gaspar et al., 1989)	Synthesis of norepinephrine through oxidative hydroxylation of dopamine (Spencer et al., 2002).	Anoxia (Miguel et al., 2018). Inflammatory cytokines (Myint and Kim, 2014).	Methylphenidate (Millan et al., 2000; Ihalainen et al., 2001; Wilens, 2008). Amphetamines (Faraone, 2018). Guanfacine (Alamo et al., 2016). Atomoxetine (Garnock-Jones and Keating, 2009). Clonidine (Cutler et al., 2020).
			NET1/SLC6A2	Presynaptic membrane (Borodovitsyna et al., 2017).	Brain cortex (Borodovitsyna et al., 2017).	Reuptake of norepinephrine and epinephrine into presynaptic nerve terminals is a regulator of norepinephrine homeostasis (Borodovitsyna et al., 2017).		
			ADRA2C	Presynaptic membrane (Ihalainen et al., 2001).	Central noradrenergic neurons (Ihalainen et al., 2001).	Regulates the catecholamine-induced inhibition of adenylate cyclase through the action of G proteins (Ihalainen et al., 2001).		
Acetylcholine	Dopaminergic neurons, GABAergic interneurons, and pyramidal neurons (Bates et al., 2014).	It mediates learning, attention, and memory (Viggiano et al., 2004).	CHRNA7	Both presynaptic and glial cell membranes (Arroyo-Jim nez et al., 1999; Klink et al., 2001; Gotti et al., 2006; Bates et al., 2014).	Hippocampus (Arroyo-Jim nez et al., 1999; Klink et al., 2001; Gotti et al., 2006).	It facilitates dopamine release in the nucleus accumbens and the striatum (Picciotto et al., 1998; Grady et al., 2002). It regulates the dopamine transporter gene transcription and function (Li et al., 2004; Parish et al., 2005). It facilitates the transmission of GABA from the synapse to the postsynaptic membrane and glial cells (Bates et al., 2014).	None	None
			CHRNA4	Postsynaptic and synapse (Albuquerque et al., 2009).	Basal ganglia, amygdala, and ventral tegmental area (Albuquerque et al., 2009).	Regulates the release of dopamine (Albuquerque et al., 2009).		
Glutamate	GABAergic, cholinergic, and dopaminergic neurons (Dalezios et al., 2002; Wang and Pickel, 2002; Loonen et al., 2017).	Regulate brain development, modulation of neuronal activity, regulation of dopamine signaling, synaptic plasticity, memory formation, and learning (Suh et al., 2018).	GRM1	Postsynaptic membrane (Suh et al., 2018).	Basal ganglia and cerebellum (Berthele et al., 1999).	Synthesis of GABA and bidirectional regulation of dopamine signaling (Loonen et al., 2017).	Inflammatory cytokines (Myint and Kim, 2014). Manganese (Sidoryk-Wegrzynowicz, 2014). Ethanol (Gibson et al., 2000).	Methylphenidate (Park et al., 2014).
			GRM5	Postsynaptic membrane (Suh et al., 2018).	Basal ganglia and cerebellum (Berthele et al., 1999).	Critical for inhibitory learning mechanisms (Xu et al., 2009).		
			GRM7	Presynaptic membrane (Kinoshita et al., 1998).	Amygdala, hippocampus, and the locus coeruleus (Kinoshita et al., 1998).	Presynaptic regulator of neurotransmission in the mammalian central nervous system (Kinoshita et al., 1998).		
			GRM8	Presynaptic membrane (Suh et al., 2018).	Hippocampus (Ferraguti et al., 2005)	Adjusting the activity of GABAergic interneurons (Ferraguti et al., 2005).		
GABA	Glutamatergic neurons (Loonen et al., 2017).	Regulates dopamine tone (Loonen et al., 2017).	GAD1	Presynaptic membrane (Pinal and Tobin, 1998).	Frontal cortex and hippocampus (Pinal and Tobin, 1998)	Modulate behavioral inhibition and self-control (Silveri et al., 2013).	Inflammatory cytokines (Myint and Kim, 2014). Manganese (Sidoryk-Wegrzynowicz, 2014).	Methylphenidate (Freese et al., 2012).

ADHD, attention-deficit/hyperactive disorder; ADRA2C, Alpha-2C-adrenergic receptor; CHRNA4, cholinergic receptor, neuronal nicotinic, alpha polypeptide 4; CHRNA7, cholinergic receptor, neuronal nicotinic, alpha polypeptide 7; DRD2, dopamine receptor D2; DRD4, dopamine receptor D4; DAT1, dopamine transporter 1; DBH, dopamine beta-hydroxylase; GABA, γ-aminobutyric acid; GAD1, glutamate decarboxylase 1; GRM1, glutamate receptor metabotropic 1; GRM5, glutamate receptor metabotropic 5; GRM7, glutamate receptor metabotropic 7; GRM8, glutamate receptor metabotropic 8; NET1, neuroepithelial cell transforming gene 1; HTR1A, 5-hydroxytryptamine receptor 1A; HTR2A, 5-hydroxytryptamine receptor 2A; HTR1B, 5-hydroxytryptamine receptor 1B; KO, knockout; SLC6A4, solute carrier family 6 (neurotransmitter transporter, serotonin), member 4; SLC6A2, solute carrier family 6 (neurotransmitter transporter, serotonin), member 2; TPH2, tryptophan hydroxylase 2.

**TABLE 2:** Genes related to ADHD in animal models and modulators.

Gene/animal model	Modulator	Animal model for ADHD	Phenotype	Key findings
***DRD1***	SCH 23390 is antagonist (Eagle et al., 2011).	None	None	None
***DRD2***	Sulpiride is antagonist (Eagle et al., 2011). Bromocriptine is agonist (Rokem et al., 2012).	None	None	None
***DRD4***	PNU-101387G and PD 168,077 are agonists (Nayak and Cassaday, 2003; Avale et al., 2004). RO-10-5824 is a selective agonist (Powell et al., 2003). Antagonists include CP-293,019, L-745,870, U-101,958, and S-18126 (Zhang et al., 2002).	KO mice (Avale et al., 2004).	Hyperactive (Avale et al., 2004).	Hyperactivity and impaired behavioral inhibition (Avale et al., 2004).
		KO mice (Helms et al., 2008)	None	Lack of DRD4 is not adequate to cause impulsivity (Helms et al., 2008).
		Knock-in mice (Bonaventura et al., 2017).	ADHD (Bonaventura et al., 2017).	DRD4 regulates corticostriatal glutamatergic neurotransmission and enhanced DRD4 activity is related to ADHD (Bonaventura et al., 2017).
		Transgenic mice (DRD4−/− and DRD4±) (Thomas et al., 2009)	None	Lowered DRD4 expression increases extracellular levels of glutamate neurotransmission in the striatum of DRD4−/− mice (Thomas et al., 2009).
		Viral expression of the DRD4 7R allele in the prefrontal cortex of D4RD knockout mice (Qin et al., 2016).	ADHD (Qin et al., 2016)	*DRD4* 7R allele causes over-suppression of NMDA receptor function in the prefrontal cortex (Qin et al., 2016).
***DAT1***	None	KO mice (Pogorelov et al., 2005).	Anxiety, novelty seeking, and stereotypic activation (Pogorelov et al., 2005).	*DAT* KO is related to anxiety, novelty seeking, and stereotypical-perseverative spectrum (Pogorelov et al., 2005).
		KO mice (Rodriguiz et al., 2004).	Abnormal social interaction (Rodriguiz et al., 2004).	*DAT* KO displays abnormal social interaction (Rodriguiz et al., 2004).
		KO mice (Kurzina et al., 2021).	Hyperactivity and impaired cognition (Kurzina et al., 2021).	*DAT* KO exhibit impaired learning the cognitive task and hyperactivity (Kurzina et al., 2021).
		DAT hypofunctional mice (DAT±) (Mereu et al., 2017).	Hyperactivity, attentional, and impulsivity deficits (Mereu et al., 2017).	*DAT* hypofunctional mice exhibited hyperactivity, attentional, and impulsivity deficits. These mice showed decreased expression of Homer1a in the prefrontal cortex and amphetamine treatments rescued hyperactivity and cognitive deficits (Mereu et al., 2017).
		KO mice (Reinwald et al., 2022).	Motor hyperactivity and compulsive-like features (Reinwald et al., 2022).	*DAT*-KO rats had volume loss in the dorsal striatum, negatively correlating with cerebellar volume increase. They also have altered striato-cerebellar and prefrontal-midbrain circuits (Reinwald et al., 2022).
***TPH2***	None	*Tph2* null mutant mouse (Akhrif et al., 2021).	ADHD (Akhrif et al., 2021).	*Tph2* null mutant mouse reproduces the *TPH2* G-703T-dependent ADHD phenotype in humans (Akhrif et al., 2021).
		*TPH2*-deficient mice (Waider et al., 2011).	Anxiety-like behavior (Waider et al., 2011).	*TPH2*-deficient mice show anxiety-like behavior (Waider et al., 2011).
***HTR1A***	None	None	None	None
***HTR1B***	None	KO mice (Bouwknecht et al., 2001).	Disinhibition, hyperactivity, and increased aggression (Bouwknecht et al., 2001).	*5HT1B* KO mice exhibited behavioral disinhibition, hyperactivity, and increased aggression (Bouwknecht et al., 2001).
***SLC6A4***	None	None	None	None
***HTR2A***	None	None	None	None
***DBH***	None	None	None	None
***NET1/SLC6A2***	None	None	None	None
***ADRA2A***	None	None	None	None
***ADRA2C***	None	None	None	None
***CHRNA4***	None	None	None	None
***CHRNA7***	3-2,4 dimethoxybenzylidene anabaseine is agonist (Martin et al., 2004).	None	None	None
***GAD1***	None	None	None	None
***GRM1***	None	None	None	None
***GRM5***	None	None	None	None
***GRM7***	None	None	None	None
***GRM8***	None	None	None	None
***TARBP1***	None	None	None	None
***ADGRL3/LPHN3***	None	Mice null for *Lphn3* (Wallis et al., 2012).	Hyperactive (Wallis et al., 2012).	Alteration in genes including dopamine and serotonin receptors and transporters fluctuations in neurotransmitter metabolism genes and in neural developmental genes. Elevated levels of dopamine and serotonin in the dorsal striatum (Wallis et al., 2012).
***FGF1***	None	None	None	None
***MAOA***	None	None	None	None
***BDNF***	None	None	None	None
***SNAP25***	None	None	None	None
***STX1A***	None	None	None	None
***ATXN7***	Atomoxetine is a blocker (Dela Peña et al., 2019).	Atxn7 overexpressing (Atxn7 OE) mice (Dela Peña et al., 2019).	Hyperactivity and impulsivity (Dela Peña et al., 2019).	There was overexpression of the *Atxn7* gene and protein in the prefrontal cortex and striatum of the *Atxn7* OE mice. Atomoxetine rescued hyperactivity and impulsivity (Dela Peña et al., 2019).
***SORCS2***	None	SorCS2-deficient mice (Olsen et al., 2021).	ADHD (Olsen et al., 2021).	Impaired dopaminergic circuit function in the ventral tegmental area (Olsen et al., 2021).
**Spontaneously Hypertensive Rat (SHR)**	None	Spontaneously Hypertensive Rat (SHR)	ADHD (Mill et al., 2005b).	There was no difference in terms of the expression of *Drd2* or *Drd4*, but some variations were found in *Dat1* (Mill et al., 2005b).
		Spontaneously Hypertensive Rat (SHR)	ADHD (Satoh et al., 2018).	Hypofunction of the DRD1 pathways of GABAergic interneurons in the anterior cingulate cortex (Satoh et al., 2018).
***CK1*δ**	None	Mice overexpressing CK1δ (CK1δ OE) (Zhou et al., 2020).	ADHD (Zhou et al., 2020).	*CK1*δ OE impairs transcriptional homeostasis in the striatum including *Drd1a* versus Drd2 neurons (Zhou et al., 2020).

ADHD, attention-deficit/hyperactive disorder; ADGRL/LPHN3, atrophilin-3; ADRA2C, alpha-2C-adrenergic receptor; ATXN7, ataxin 7; BDNF, brain-derived neurotrophic factor; CHRNA4, cholinergic receptor, neuronal nicotinic, alpha polypeptide 4; CHRNA7, cholinergic receptor, neuronal nicotinic, alpha polypeptide 7; DRD2, dopamine receptor D2; DRD4, dopamine receptor D4; DAT1, dopamine transporter 1; DBH, dopamine beta-hydroxylase; GABA, γ-aminobutyric acid; GAD1, glutamate decarboxylase 1; GRM1, glutamate receptor metabotropic 1; GRM5, glutamate receptor metabotropic 5; GRM7, glutamate receptor metabotropic 7; GRM8, glutamate receptor metabotropic 8; FGF1, fibroblast growth factor 1, neuroepithelial cell transforming gene 1; HTR1A, 5-hydroxytryptamine receptor 1A; HTR2A, 5-hydroxytryptamine receptor 2A; HTR1B, 5-hydroxytryptamine receptor 1B; KO, knockout; MAOA, monoamine oxidase A; SLC6A4, solute carrier family 6 (neurotransmitter transporter, serotonin), member 4; SLC6A2, solute carrier family 6 (neurotransmitter transporter, serotonin), member 2; SNAP25, synaptosome associated protein 25; STX1A, Syntaxin 1A; SORCS2, Sortilin Related VPS10 Domain Containing Receptor 2; TPH2, tryptophan hydroxylase 2; TARBP1, TAR (HIV-1) RNA Binding Protein 1.

### Genetic mechanisms and their interactions with environmental factors

Attention-deficit/hyperactive disorder is a heterogeneous and highly heritable disorder resulting from complex gene–gene and gene–environment interactions (Smalley, 1997; Faraone, 2004; Faraone et al., 2005; Banaschewski et al., 2010). Multiple genetic studies support a strong genetic contribution to the disorder’s phenotype. Biological parents and siblings of patients with ADHD have a two- to eight-fold risk of having it too (Biederman et al., 1990, 1992). The heritability is estimated to be 0.76 for childhood ADHD (Faraone et al., 2005). The usage of genome-wide association studies has found many variants that are implicated in ADHD, including common and rare ones from a broad range of genes related to neurotransmission and neurodevelopment (Akutagava-Martins et al., 2013). The results from the copy number variations (CNVs) tests consolidate the idea that, alongside common variants, rare variants are involved in ADHD etiology (Williams et al., 2012; Martin et al., 2015). This includes rare recurrent variants in non-coding regions as revealed by the whole genome sequencing from the children of both African American and European American ancestry (Liu et al., 2021a). Furthermore, miRNA studies based on animal models, cell lines, or human subjects show the emerging trend of miRNA in ADHD (Srivastav et al., 2018).

**FIGURE 1:**
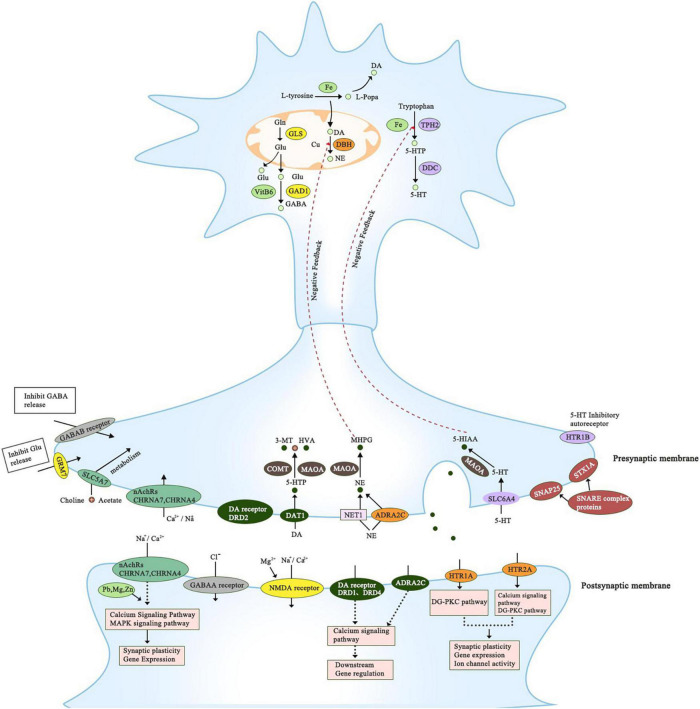
This figure summarizes all genes that have been reported to associate with ADHD. It includes genes that belong to the proposed pathways; dopaminergic, serotonergic, adrenergic, cholinergic, glutamatergic, and GABAergic. The precise location of the gene is presented; axon, presynaptic and postsynaptic compartments. All of the genes have a significant role in regulating dopamine neurotransmission directly or indirectly.

Multiple genes have been reported to associate with ADHD in children. All reported genes have a significant association with neurotransmission and neurodevelopment which can explain ADHD etiology and pathophysiology (Figure 1). They fall under six pathways: dopaminergic, serotonergic, adrenergic, cholinergic, glutamatergic, and GABAergic, and can interact with environmental factors as shown in Table 1. Some of the reported genes in humans have evidence from animal models while others don’t (Table 2).

#### Genes and microRNAs related to dopaminergic pathway

Dopamine exerts its effects in the frontal cortex through GABAergic neurons (Saunders et al., 2015; Kramer et al., 2020), noradrenergic (Zhu, 2018), as well as serotonergic neurons (Di Matteo et al., 2008). Dopamine neurotransmission comprises numerous dopamine signaling pathways: mesolimbic (dopaminergic neurons projecting from the ventral tegmental area to the nucleus accumbens), neocortical (dopaminergic neurons projecting from the ventral tegmental area to the frontal cortex), and mesostriatal (dopaminergic neurons projecting from the ventral tegmental area to the caudate putamen) (Chen et al., 2016). Signals transmitted by dopamine regulate emotion, reward, locomotion, complex behavior, and cognition (Snyder, 2011). Dysfunction of dopamine signaling in the forebrain leads to inappropriate or deficient attention (Huang et al., 2015). Animal models of ADHD showed dysregulation of dopamine functions and abnormal behaviors which were normalized upon administration of stimulant medications (Russell, 2011). Some studies based on children with ADHD identified frontal cortical regions, which are rich in dopamine, as sites related to ADHD (McAlonan et al., 2007, 2009). Additionally, several studies have shown that the dopamine receptor density in several brain regions of ADHD patients is lower compared to healthy individuals (Jönsson et al., 1999; Medin et al., 2013). Notably, it has been revealed that it is the elevation of the frontal cortical dopamine levels and not norepinephrine or serotonin that acts as a convergent mechanism for the paradoxical effects of ADHD psychostimulants therapies (Harris et al., 2022). ADHD is associated with higher mortality and lowered estimated life expectancy by adulthood and some of the dopaminergic SNP has been reported to relate to 5- and 2-year reductions in estimated life expectancy (Barkley et al., 2019). Consequently, the dopaminergic pathway has a crucial role to play in the occurrence of ADHD. Genes reported in this pathway include *DRD1, DRD2*, *DRD4*, and *DAT1.*

##### DRD1

*DRD1* gene encodes for DRD1 (dopamine receptor D1) receptor. DRD1 is found in the postsynaptic membrane where it regulates the postsynaptic transmission of dopamine. It is expressed in excitatory neurons in the prefrontal cortex (Navandar et al., 2021). It is a G-protein coupled receptor (GPCR) that activates adenylyl cyclase after dopamine binds to it (Undieh, 2010). After the activation of the adenylyl cyclase, there is a generation of the cyclic AMP (cAMP) which in turn activates protein kinase A and cAMP-mediated gene expression (Sassone-Corsi, 2012). There is an association between *DRD1* polymorphisms and childhood ADHD according to the case-control study conducted in Spain (Ribasés et al., 2012). Another study which was conducted among 232 families revealed that DRD1 polymorphisms are more related to the inattention phenotype (Luca et al., 2007). Likewise, another case-control study revealed a relationship between *DRD1* polymorphisms and ADHD (Bobb et al., 2005). It has been shown that the hypofunction of DRD1-mediated regulation of GABAergic inhibitory synaptic transmission onto layer V pyramidal cells of the anterior cingulate cortex might play a role in the pathophysiology of ADHD as demonstrated in spontaneously hypertensive rats (Satoh et al., 2018).

##### DRD2

*DRD2* gene encodes for DRD2 (dopamine receptor D2) receptor. DRD2 is highly expressed in the prefrontal cortex, responsible for locomotion, reward, reinforcement, memory, and learning (Usiello et al., 2000). DRD2 is a GPCR that inhibits adenylyl cyclase, found in the dopaminergic synapse. DRD2 is found in both presynaptic and postsynaptic membranes, and facilitates the reuptake of dopamine from the synapse to the presynaptic membrane *via* DAT1. When dopamine binds to the DRD2, it inhibits adenylate cyclase and thus decreasing intracellular concentrations of cAMP, hence the change in intracellular and extracellular concentrations of ions causing hyperpolarization of cell membrane which affects the transmission of the impulse. Meanwhile, it can also activate mitogen-activated protein kinase (MAPK) which in turn activates the calcium signaling pathway which influences synaptic plasticity, affects downstream gene regulation, long- and short-excitability as well as cell death or survival. It is also responsible for postsynaptic neurotransmission of NE (Robbins et al., 1988).

There are some evidence from human beings that relate *DRD2* polymorphisms and ADHD (Supplementary Table 1). *DRD2* genetic predisposition is associated with aggressive behavior, memory dysfunction, and ADHD (Fernandez et al., 2022). *DRD2* Taq1A (rs1800497) (Neville et al., 2004; Pan et al., 2015) has been reported to relate to DRD2 expression levels (Laakso et al., 2005). *DRD2-12* (*rs7131465*) is related to a higher risk for ADHD and autism spectrum disorder overlap (Mariggiò et al., 2021). The intronic DRD2 SNPs (rs1079727, rs1079595, and rs1124491), and SNP in the 3’-UTR (rs1800497) have been linked with ADHD in males (Nyman et al., 2007). T allele of rs1800497 was also identified as a risk of ADHD in a meta-analysis (Pan et al., 2015). The interaction of *ADORA2A* and *DRD2* genes has been reported to be responsible for anxiety disorders in ADHD children and adolescence (Fraporti et al., 2019). There is a significant interaction effect of DRD2 rs1800497 and lead exposure on the cortical thickness of the prefrontal cortex in ADHD according to the case-control study (Kim et al., 2018a). Currently, there is no *DRD2* animal model for ADHD.

##### DRD4

*DRD4* gene encodes DRD4 (dopamine receptor D4) receptor. This receptor is a GPCR that inhibits adenylyl cyclase, found in the dopaminergic synapse. DRD4 is expressed in the prefrontal cortex including the anterior cingulate and orbitofrontal cortex, which are regions predominantly affected in ADHD (Noaín et al., 2006; Leung et al., 2017). DRD4 is located in the postsynaptic membrane and is responsible for the postsynaptic transmission of dopamine. Similar to DRD2, when dopamine binds to the DRD4, it inhibits adenylate cyclase and thus decreases intracellular concentrations of cAMP, hence change in intracellular and extracellular concentrations of ions causing hyperpolarization of cell membrane which affects the transmission of the impulse. Meanwhile, it can also activate MAPK which in turn triggers the calcium signaling pathway which influences synaptic plasticity, affects downstream gene regulation, long-and-short excitability, as well as cell death or survival.

There are some evidence both from humans (Supplementary Table 1) and animal models (Table 2) to support the relationship between *DRD4* gene polymorphisms and ADHD. However, it is worth noting that most of the animal models are knockouts of which the majority do not replicate the full manifestation of ADHD as seen in patients. The variable number of tandem repeats (VNTR) in exon 3 of the *DRD4* gene has been implicated in the etiology of ADHD (Chang et al., 1996; Bidwell et al., 2011). Interestingly, different populations have different risk alleles, for instance, the 2-repeat allele (2R) has been noticed in the Han Chinese (Chang et al., 1996) and Koreans (Hong et al., 2018), 4-repeat allele (4R) for Asians while 7-repeat allele (7R) for white Europeans and Americans (Faraone et al., 1999; Hawi et al., 2000; Shahin et al., 2015; Tabatabaei et al., 2017). A haplotype of markers: allele 2 of the 120 bp duplication, 616 C/G substitutions, and 521 C/T substitutions of the C allele can confer a predisposition to ADHD (Mill et al., 2003). *DRD4* 7R allele is related to reduced prefrontal gyrification in children with ADHD (Palaniyappan et al., 2019). *DRD4* 2R allele can affect the default mode network, the executive control network, and the sensorimotor network in the prefrontal cortex circles (Qian et al., 2018a). According to functional magnetic resonance imaging, *DRD4* polymorphisms are associated with abnormality in the frontal–striatal–cerebellar loop among ADHD children (Qian et al., 2018b). The sensitive allele repeats in the *DRD4* gene have been suggested as a prognostic factor (Tabatabaei et al., 2017).

Besides, *DRD4*-rs916457 has been linked with abnormal neuropsychological tasks outlining working memory and perceptual organization in ADHD patients (Cervantes-Henriquez et al., 2021). The *DRD4*-521 C/T SNP regulates the transcription rate and expression for *DRD4* which affects the level of dopamine in the brain (Zhang et al., 2021a). DRD4 and α2AR co-localize in cortical pyramidal neurons to form α_2*A*_R-D_4_R heteromers which play a pivotal role in catecholaminergic signaling in the brain cortex and are potential targets for ADHD pharmacotherapy (Casadó-Anguera et al., 2021). SNPs in *DRD*4 relate to distinct domains of ADHD severity (Cervantes-Henríquez et al., 2022). The 7R allele of the *DRD4* gene is more prevalent in sluggish cognitive cases than in ADHD cases (Bolat et al., 2020). It has been reported that the interaction between the methylation of CpG sites of *DRD4* (CpG26 and CpG28) and phthalate metabolite levels can affect the attention level in ADHD patients (Kim et al., 2021). It has been shown that *Drd4* knockout mice demonstrate hyperactivity and impaired behavioral inhibition signifying that intact Drd4 signaling during development is essential for the development of ADHD phenotype (Avale et al., 2004). In another *Drd4* knockout mice study, it has been revealed that a lack of DRD4 is not adequate to cause impulsivity (Helms et al., 2008). Viral expression of the DRD4 7R allele in the prefrontal cortex of *Drd4* knockout mice exhibits augmented exploratory and novelty-seeking behaviors, mimicking human ADHD (Qin et al., 2016). This DRD4 7R allele causes over-suppression of NMDA receptor function in the prefrontal cortex (Qin et al., 2016). *Drd4* knock-in mice demonstrate augmented DRD4-mediated dopaminergic regulators of corticostriatal transmission which is associated with ADHD (Bonaventura et al., 2017). Transgenic mice (*DRD4*^–/^*^–^* and *DRD4*±) showed that reduced DRD4 expression increases extracellular levels of glutamate neurotransmission in the striatum of *DRD4*^–/^*^–^* mice (Thomas et al., 2009).

In regard to pharmacology, children carrying *DRD4* homozygous long 7-repeat allele showed better behavioral response to methylphenidate (Naumova et al., 2019). A recent meta-analysis suggested that *DRD4* 48 bp VNTR variants can be considered as ADHD biomarkers to support the diagnosis and to predict methylphenidate response in children (Bonvicini et al., 2020). Besides, the interaction between methylation of CpG7 of *DRD4* and prenatal maternal stress has been suggested as a possible predictor of the methylphenidate response in youth with ADHD (Kim et al., 2018b). *DRD4* 4R showed a significant association with methylphenidate response according to one meta-analysis (Myer et al., 2018). Children homozygous for the DRD4 7R allele showed a better response to methylphenidate according to the placebo-controlled trial (Naumova et al., 2019). The interaction between genetic polymorphisms of *DRD4* and organophosphate pesticide exposure as well as oxidative stress increases the risk of ADHD in children (Chang et al., 2018). It has been shown that fetal methylphenidate exposure prompts ADHD-like phenotypes and reduces *Drd2* and *Slc6a3* expression levels in mouse descendants (Aoki et al., 2021).

##### DAT1

*DAT1* gene encodes for dopamine transporter 1 (DAT) which is located in the presynaptic membrane (Diamond, 2007). It is more expressed in the striatum and prefrontal cortex (Diamond, 2007). It mediates the reuptake of dopamine from the synapse and primary regulator of dopaminergic neurotransmission (Ernst et al., 1999). Reduction in expression of *DAT1* can reduce reuptake and increase the metabolism of dopamine (Ernst et al., 1999). There are some evidence both from humans (Supplementary Table 1) and knockout animal models (Table 2) to support the relationship between *DAT1* gene polymorphisms and ADHD or some of the ADHD phenotypes. *DAT1* VNTR polymorphism can modulate working memory in ADHD children (Pineau et al., 2019). The 6R and 10R have been considered as risk alleles for childhood ADHD while 9R has been associated with adulthood ADHD (Chen et al., 2003; Mill et al., 2005a; Brown et al., 2011; Agudelo et al., 2015). The 10R/10R genotype of *DAT1* VNTRs is related to ADHD in Korean children (Hong et al., 2018). The haplotype rs27048 (C)/rs429699 (T) has been suggested as a genetic marker for the inattentive ADHD subtype in the Chinese population (Shang et al., 2011). Likewise, rs27072 polymorphism is related to inattention and hyperactivity/impulsivity (Ouellet-Morin et al., 2008). It has been reported that there is no association between VNTR polymorphism of *DAT1* and *DRD4* genes and ADHD among Indonesian children based on case-control study (Thursina et al., 2020). *DAT1* 9 R carriers have a higher likelihood of high traffic risk behavior in case only they also have ADHD symptoms (Tokko et al., 2022).

*DAT*-knockout rats show impairment in learning the cognitive task, however, their hyperactivity does not prevent the capability to learn a non-spatial cognitive task in the appearance of novel stimuli (Kurzina et al., 2021). Importantly, DAT genetic hypofunction in mice produced alterations consistent with ADHD, but not with schizophrenia or bipolar disorders (Mereu et al., 2017) in one study, but it was shown in another study that DAT silencing in the rat produces abnormalities in the prefrontal-midbrain and in striato-cerebellar circuits leading to motor hyperactivity and compulsive-like behaviors applicable for ADHD, schizophrenia, and obsessive-compulsive disorder (Reinwald et al., 2022). *DAT* hypofunctional mice exhibit hyperactivity, attentional and impulsivity, and decreased expression of Homer1a in the prefrontal cortex, notably, amphetamine treatments rescued hyperactivity and cognitive deficits (Mereu et al., 2017). *DAT* knockout also exhibits anxiety, novelty seeking, and stereotypical-perseverative spectrum (Pogorelov et al., 2005). DAT-*Cnr2* conditional knockout mice (mice lacking cannabinoid CB2 receptors) in midbrain dopaminergic neurons demonstrated a hyperactivity phenotype of ADHD (Canseco-Alba et al., 2021). *P35* knockout mice (ADHD animal model) display reduced dopamine uptake and cell surface DAT expression levels in the striatum. *DAT1* gene 5′-UTR methylation has been suggested as a new approach for the exploration of an epigenetic biomarker in ADHD diagnosis. Paternal nicotine exposure prompts hyperactivity in C57BL/6 mice next-generation through down-regulating the expression of DAT which results from hyper-methylation of DAT (Zhang et al., 2020).

There are several DAT postnatal epigenetic modifiers revealed in rat brain (Green et al., 2019). *Dnmt1*, *Dnmt3a*, *Dnmt3b*, and *Hdac2* exhibit age-related decreases in DNA mRNA expression while *Hdac5* and *Hdac8* demonstrate increased mRNA expression with age (Green et al., 2019). Besides, there is a protein enhancement of acetylated histone 3 at lysines 9 and 14 as well as the transcription factors (dopaminergic) Nurr1 and Pitx3 within the DAT promoter according to age (Green et al., 2019). The loss of the *Slc6a8* (creatine) gene in DAT (Slc6a3) expressing cells leads to hyperactivity but sparing motor function as shown in DAT-specific Slc6a8 knockouts (dCrt-/y) (Abdulla et al., 2020). SUMOylation plays a critical role in regulating DAT protein hemostasis, dopamine uptake, and dopamine signaling in neurons; therefore, DAT SUMOylation can be the potential therapeutic target in regulating DAT stability and dopamine clearance in ADHD (Cartier et al., 2019).

##### MicroRNAs and dopaminergic pathway

There is a close relationship between levels of circulating miRNAs and ADHD. Wu et al. (2010) studied spontaneously hypertensive rats whereby they discovered miRNA let-7d as an important regulator of galectin-3 expression in the prefrontal cortex which is the affected area in ADHD. They noticed low levels of galectin-3 expression in the prefrontal cortex, which is important for dopamine metabolism (Wu et al., 2010). Then, they investigated serum miRNA let-7d in children with ADHD in comparison with controls, whereby, they found high circulating miRNA let-7d in ADHD subjects than in controls (Wu et al., 2015). Kandemir et al. (2014) found decreased levels of miR18a-5p, miR22-3p, miR24-3p, miR106b-5p, and miR107 in ADHD subjects compared with controls. Additional reported miRNAs include miR-125b-5p, miR-155-5p, miR-138-1, miR-296, miR-34c, miR-34b-3p, miR-34c-3p, miR-let-7d, miR-96, miR-641, miR-30b-5p, miR-1301, miR-6070, and miR-5692b (Wu et al., 2010, 2015, 2017; Németh et al., 2013; Sánchez-Mora et al., 2013; Kandemir et al., 2014; Šerý et al., 2015; Garcia-Martínez et al., 2016; Aydin et al., 2019). MiR-384-5p overexpression increases phosphorylation of the cAMP response element-binding protein (CREB) and reduces DAT levels in the prefrontal cortex of spontaneously hypertensive rats, whereas, miR-384-5p suppression increases DRD1 and decreases DAT and CREB protein levels (Xu et al., 2019). High levels of miR-132-3p have been identified in ADHD children (Coskun et al., 2021). More details about microRNAs and their associated gene(s) are given in Supplementary Table 2.

###### Dopaminergic pathway and environmental/social interactions

Iron helps in the homeostasis of the hemoglobin structure, antioxidants, genetic repair, and in particular, central nervous system function (Chen et al., 2013). To date, four meta-analyses have evaluated peripheral iron levels in children with ADHD (Biederman, 2005; Bonvicini et al., 2016; Wang et al., 2017; Tseng et al., 2018). The most recent one indicated that children with iron deficiency have more severe symptoms as compared to those without iron deficiency. Lower serum ferritin levels and iron deficiency are the risk factor for ADHD which can be explained by the following possible pathophysiological mechanisms. First, iron deficiency may impair the production of dopamine which has a prominent role in the pathophysiology of ADHD (Biederman, 2005; Burhans et al., 2005; Daubner et al., 2011). Therefore, iron deficiency disrupts dopamine production as shown in several animal studies (Youdim et al., 1983; Hyacinthe et al., 2015; Ranganath and Jacob, 2016). This dysregulation of dopaminergic neurons may further result in multiple frontal dysfunctions that mimic the symptoms of ADHD (Ghorayeb et al., 2019). Second, lower ferritin levels may provide indirect evidence of elevated oxidative stress (Finazzi and Arosio, 2014), and oxidative stress has also been reported in patients with ADHD (Adisetiyo et al., 2014). This increased oxidative stress burden may disturb neurodevelopmental trajectories and gene functions potentially predisposing to the onset of ADHD (Adisetiyo et al., 2014). Despite these evidence, iron supplementation solely does not improve ADHD symptoms (Sever et al., 1997).

Zinc is a very important cofactor for more than 100 enzymes and is required for modulating melatonin and dopamine. Dopamine plays a major role in the pathogenesis of ADHD as has been described before (Lepping and Huber, 2010). Animal and human studies indicated that zinc deficiency is associated with an increased prevalence of hyperactivity (Bettger et al., 1979). Some studies have suggested that zinc is significantly deficient in children with ADHD as compared to controls (Gao et al., 2014). Furthermore, the hair zinc level was positively correlated with inattention and hyperactivity (Tippairote et al., 2017). Lower copper/zinc and lead/zinc ratios and marginally higher zinc and magnesium levels in the hair of ADHD children have been reported (Tippairote et al., 2017). Zinc increased the affinity of methylphenidate to the dopamine transporter, however, zinc supplementations solely could not improve ADHD (Akhondzadeh et al., 2004; Arnold et al., 2011; Tippairote et al., 2017).

Ethanol affects dopamine release and impairs the binding of glutamate to mGluRs in the postsynaptic membrane (Gibson et al., 2000). Parental alcoholism presents an increased risk of offspring with ADHD (Knopik et al., 2005; Eichler et al., 2018). Additionally, rats exposed to ethanol prenatally showed attention deficits that are similar to children with ADHD than controls (Hausknecht et al., 2005). Children whose mothers smoked during pregnancy had a higher incidence of ADHD than controls (Schmitz et al., 2006; Neuman et al., 2007). Animal studies also showed that prenatal nicotine exposure increases locomotor activity in mice (Paz et al., 2007). Neonatal anoxia/hypoxia is another environmental risk that has been reported (Lou, 1996). Neonatal anoxia caused a sequence of acute and persistent neurochemical changes in rat monoaminergic systems as well as transient hyperactivity and spatial memory impairment that persisted into adulthood (Miguel et al., 2018). Additionally, animals exposed to hypoxia presented with impaired executive function associated with tissue atrophy and dopaminergic disturbance in the prefrontal cortex (Miguel et al., 2018).

Exposure to pyrethroid pesticides has been linked with ADHD both in human beings and animals. There are associations between pyrethroid insecticide use and urinary 3-phenoxybenzoic acid concentrations among Korean preschool-age boys (Lee et al., 2020). Mice exposed to pyrethroids demonstrated ADHD symptoms (Richardson et al., 2015) and abnormal dopamine neurotransmission (Bloomquist et al., 2002; Elwan et al., 2006). Maltreatment and emotional trauma correlate significantly with childhood mental disturbances. Famularo et al. (1992) discovered that children who had suffered maltreatment exhibited significantly greater incidences of ADHD than the controls. McLeer et al. (1994) found the most frequent diagnosis of sexually abused children to be ADHD in about 46% of cases. Noteworthy, among children without a strong genetic vulnerability, environmental risk factors become important, however, among children with a substantial genetic vulnerability, the impact of environmental risk factors becomes less important (Rowland et al., 2018). Nevertheless, more studies are needed to evaluate whether other environmental risk factors and parental history can independently predict ADHD occurrence. Rat pups reared in social isolation displayed a variety of behavioral changes, including hyperactivity, impulsivity, aggression, anxiety, and learning and memory deficits (Dalley et al., 2002; Koike et al., 2009).

#### Genes related to serotonergic pathway

Serotonin exerts its effects in the frontal cortex through GABAergic neurons (Lladó-Pelfort et al., 2012). Serotonin can also interact with dopaminergic, glutamatergic, and acetylcholine neurons (Lucki, 1998; Lladó-Pelfort et al., 2012; Zuo et al., 2015). Signals transmitted by serotonin regulate cognition, behavior, and immunity (Puig and Gulledge, 2011). A disruption in serotonergic development can change the brain’s function, which results in behavioral changes such as depression, anxiety, impulsivity, violence, and irregular appetite (Lucki, 1998; Sheehan et al., 2007). Reduction in serotonergic function has been linked to the impulsive subtype, which is supported by biochemical and pharmacological evidence. Relatively low platelet serotonin levels have been reported in patients with ADHD (Rapoport et al., 1974; Gainetdinov et al., 1999). Furthermore, the hyperactive state of *DAT*-KO mice was attenuated when fluoxetine (a selective serotonin re-uptake inhibitor) was administered (Gainetdinov et al., 1999; Barr et al., 2004). The reported genes in this pathway include *TPH2*, *HTR1A*, *HTR1B*, *SLC6A4*, and *HTR2A*.

##### TPH2

*TPH2* gene encodes for TPH2 (Tryptophan hydroxylase) which is a rate-limiting enzyme in the synthesis of serotonin from tryptophan. Tryptophan hydroxylase is found in the cytosol of the presynaptic membrane and is highly expressed in the frontal cortex. Park T. W. et al. (2013) reported rs11179027 and rs1843809 as risk alleles for this disorder. Sheehan et al. (2005) also identified that there was an association between ADHD and the T allele of marker rs1843809; however, this finding was not replicated in another study (Sheehan et al., 2007). In addition, TPH2-rs4570625 and TPH2-rs11178997 were reported as risk alleles in a family study (Walitza et al., 2005), but another study performed by Shim et al. could not replicate the former (Shim et al., 2010). *TPH2* rs11179027 and *TPH2* rs1843809 alleles are linked with the genetic predisposition to ADHD in Egyptian children (Abo El Fotoh et al., 2020). The T allele (rs1843809), A allele (rs1386493), and G allele (rs1007023) have been associated with ADHD in the United Kingdom population (Brookes et al., 2006). *TPH2* (rs17110747) is related to abnormal behaviors and impaired cognition in female ADHD children according to the family based association tests (Fageera et al., 2021). *TPH2*-deficient mice show anxiety-like behavior (Waider et al., 2011). The *Tph2* null mutant mouse mimics the *TPH2* G-703T phenotype in humans (Akhrif et al., 2021). High tyrosine levels can decrease TPH2 activity and tyrosinemia type 1 presents with ADHD symptoms as shown in 8 Norwegian children (Barone et al., 2020). TPH2 methylation has been correlated with ADHD in boys at early school age in a German longitudinal cohort study (Heinrich et al., 2017). As a result, there are some conflicting results regarding *TPH2* alleles which makes it difficult to draw a conclusion. Despite these contradicting results, TPH2 has a role to play in ADHD but needs more studies.

##### 5-hydroxytryptamine receptor 1A

*HTR1A* (5-hydroxytryptamine receptor 1A) encodes the 5-HT1A receptor that binds to the endogenous neurotransmitter serotonin. This receptor is a GPCR that is found in the postsynaptic membrane and is highly expressed in limbic areas, hypothalamus, and cortex. It plays a role in the regulation of dopamine and 5-HT levels in the brain. Activation of 5-HT1A receptors stimulates the opening of G-protein-coupled inwardly rectifying K+ (GIRK) channels in CA1 pyramidal cells of the hippocampus (Brown et al., 2005), followed by the outflow of K+ ions from the intracellular to the extracellular space which hyperpolarizes the neuron (Araragi and Lesch, 2013). The activation of the 5-HT1A receptor may increase dopamine release in the medial prefrontal cortex, striatum, and hippocampus, subsequently, inhibition of the release of glutamate and acetylcholine occurs in various areas of the brain (Lucki, 1998; Zuo et al., 2015). Shim et al. (2010) reported an association between allele frequencies of *HTR1A* C1019G and ADHD. The homozygous allele C frequency was significantly higher in ADHD patients than in controls (Shim et al., 2010). Park Y. H. et al. (2013) identified rs10042486, rs1423691, and rs878567 as risk alleles of *HTR1A* for ADHD but they have not been replicated.

##### HTR2A

The *HTR2A* gene encodes HTR2A (5-hydroxytryptamine receptor 2A) which is also a GPCR for serotonin. It is found in the postsynaptic and glial cell membranes, highly expressed in the neocortex, caudate nucleus, nucleus accumbens, and hippocampus. It facilitates the reuptake of serotonin from the synapse to the glial cell and postsynaptic membrane. HTR2A plays a role in the frontal cortex, where it mediates the activation of a subpopulation of GABAergic neurons (Serretti et al., 2007). Stimulation of HTR2A activates cAMP-response element-binding protein and/or brain-derived neurotrophic factor through calcium-dependent protein kinases in the cortex, leading to complex cell modifications depending on the second messenger cascade. Few studies with a small sample size have focused on *HTR2A*, whereby in one study, *HTR2A* 452Tyr allele was found to associate weakly with this disorder (Quist et al., 2000). Inattention subtype of ADHD was reported to have a link with SNP rs7984966 (Pinto et al., 2016). Levitan et al. (2002) found that the CC genotype of the T102C variant was associated with childhood ADHD in women who later developed seasonal affective disorder.

##### HTR1B

The *HTR1B* gene encodes HTR1B (5-hydroxytryptamine receptor 1B) which is also found in the presynaptic membrane and highly expressed in the striatum, basal ganglia, and hippocampus. It inhibits the activity of adenylate cyclase and controls the release of dopamine, serotonin, and acetylcholine in the brain. Two serotonergic genes: *HTR1B* and *SLC6A4* were found to associate with ADHD according to the review (Faraone et al., 2005). Multiple SNPs that were associated with the inattentive subtype of ADHD including rs6296, rs6297, rs130060, rs6298, rs130058, and rs11568817 (Levitan et al., 2002) were once reported; however, they were not replicated in a subsequent study (Banerjee et al., 2012). Additional evidence showed that *5HT1B* knockout mice exhibit behavioral disinhibition, hyperactivity, and increased aggression (Bouwknecht et al., 2001).

##### Solute carrier family 6 member 4

Solute carrier family 6 member 4 (*SLC6A4*) is encoded by the *SLC6A4* gene. SLC6A4 is an integral membrane protein that transports the neurotransmitter serotonin from synaptic spaces into presynaptic neurons. Functional 5HTTLPR 44-bp insertion/deletion ‘long’ allele was identified as a risk allele for ADHD according to the meta-analysis (Gizer et al., 2009). The concurrence of the *SLC6A4* rs6354 GG/GT and ADRA2A rs553668GG/GA genotypes is associated with a 6.15-fold increased risk of ADHD in comparison to cases carrying the combination of *ADRA2A* rs553668 AA and *ANKK1* rs1800497 AA genotypes highlighting the role of the gene-gene interactions and polygenic effects in the occurrence of the ADHD as shown in a case-control study (Wang et al., 2021).

#### Genes related to adrenergic pathway

Norepinephrine is important for cognition, memory, regulation of stress, and uptake of lactate to the neurons. Excessive reuptake to the presynaptic membrane by transporters leads to depletion in synaptic junction hence the occurrence of ADHD clinical features. This can explain the role of norepinephrine reuptake inhibitors in patients with ADHD. Noradrenergic gene polymorphisms can affect the efficacy of methylphenidate and atomoxetine in children with ADHD according to the meta-analysis (Gul et al., 2021; Yuan et al., 2021). The reported genes include *DBH*, *DRD4, NET1*/*SLC6A2*, *ADRA2A*, and *ADRA2C*.

##### Dopamine beta-hydroxylase

Dopamine beta-hydroxylase (DBH) is responsible for the synthesis of norepinephrine through oxidative hydroxylation of dopamine and it is released into the circulation during synaptic transmissions from sympathetic neurons (Spencer et al., 2002). It is encoded by the DBH gene. Lower activities of DBH in the serum and urine of patients with ADHD have been reported (Pliszka et al., 1994), and decreased DBH levels correlated with the symptoms of this disorder in children (Rogeness et al., 1989). *DBH* KO mouse has decreased norepinephrine levels in the central neural system, which suggests the importance of this enzyme in the maintenance of normal norepinephrine functions (Cryan et al., 2001). *DBH* is highly associated with ADHD. Multiple *DBH* gene polymorphisms have been reported including rs1611115 (Kieling et al., 2008; Bhaduri et al., 2010), rs1108580 (Bhaduri et al., 2010) as well as rs6271 and rs2519152 (Tang et al., 2006). Moreover, paternal over-transmission for rs2519152 and a strong correlation between rs1611115, rs1108580, and rs2519152 and DBH enzyme activity have been identified (Bhaduri et al., 2010). The *DBH* rs1611115 is related to verbal aggression while *DRD2* rs4274224 is associated with executive functions in ADHD among adult prisoners (Fernandez et al., 2022). Nevertheless, rs1611115 and rs1108580 had no significant association with childhood ADHD according to the recent meta-analysis (Gizer et al., 2009).

##### NET1/SLC6A2

*NET1*/*SLC6A2* (neuroepithelial cell transforming gene 1) encodes for the norepinephrine transporter. It is found in the presynaptic membrane and is highly expressed in the brain cortex, responsible for the reuptake of norepinephrine and epinephrine into presynaptic nerve terminals, and is a regulator of norepinephrine homeostasis. This gene regulated intrinsic brain activity, visual memory, and attention in ADHD children (Shang et al., 2021). Biederman et al. (2008) found variants in *NET1*/*SLC6A2* associated more with ADHD in females, whereas Anney et al. (2008) reported about paternal over the transmission of risk alleles to affected individuals.

##### Alpha-2C-adrenergic receptor

Alpha-2C-adrenergic receptor (ADRA2C) is encoded by the *ADRA2C* gene and it is found in the presynaptic membrane and highly expressed in heart and central noradrenergic neurons. It regulates the catecholamine-induced inhibition of adenylate cyclase through the action of G proteins. Cho et al. (2008) found that homozygous carriers of the C allele of the Dral polymorphism in ADRA2C have a trend toward increased response time variability while individuals homozygous for the G allele at the Mspl polymorphism has a trend toward decreased response time variability. Guan et al. (2009) reported a significant association of *ADRA2C* variants with combined subtype of ADHD, but the study done by Barr et al. (2001) revealed no association between the alleles of ADRA2C genes with ADHD. Larger sample size analyses are needed to verify this.

##### ADRA2A

According to the case-control study, neurocognitive functions are affected by the interaction between blood lead levels and alpha-2A-adrenergic receptor (ADRA2A) (Choi et al., 2020). ADRA2A rs553668GG/GA and SLC6A4 rs6354 GG/GT genotypes display a 6.15-fold increase in the risk of ADHD in comparison to the cases carrying the combination of *ADRA2A* rs553668 AA and ANKK1 rs1800497 AA genotypes emphasizing the importance of the gene-gene interactions in the occurrence of the ADHD (Wang et al., 2021). Gene-environment interactions have been observed between prenatal tobacco smoking exposure and *ADRA2A* rs553668 in relation to ADHD (Wang et al., 2019). Notably, exposure to tobacco smoke has been linked with ADHD and learning disabilities in Korean children (Cho et al., 2013).

#### Genes and copy number variations related to cholinergic pathway

The neurotransmitter acetylcholine (ACh) plays a critical role in brain circuits mediating motor control, learning, attention, and memory. Cholinergic dysfunction is associated with multiple brain disorders including ADHD (English et al., 2009). There are few studies that focused on genes impacting ACh signaling as determinants of ADHD risk. Nicotinic acetylcholine receptors (nAChRs) are expressed in regions densely innervated by dopaminergic neurons (Arroyo-Jim nez et al., 1999; Klink et al., 2001; Gotti et al., 2006). The activation of presynaptic nAChRs is known to facilitate dopamine release in the nucleus accumbens and in the striatum (Picciotto et al., 1998; Grady et al., 2002). Additionally, nAChRs signaling was shown to regulate the dopamine transporter gene transcription and function, potentially affecting dopamine uptake (Li et al., 2004; Parish et al., 2005). Developmental nicotine exposure augments nicotine preference, provokes hyperactivity and risk-taking behaviors, impairs nAChR expression and function, impairs DAT function, and leads to DNA hypomethylation in frontal cortex and striatum of both first and second-generation adolescent offspring of the developmental nicotine exposure mice (Buck et al., 2019).

Two cholinergic genes (*CHRNA4* and *CHNRA7*) that encode α4 and α7 subunits of nicotinic acetylcholine receptors, respectively, have been reported (Kent et al., 2001a,b; Wallis et al., 2009; Mastronardi et al., 2016). *CHRNA4* gene (cholinergic receptor nicotinic alpha 4 subunit) encodes for the nAChR α4 subunit, which is found in the postsynaptic membrane and highly expressed in the central nervous system. CHRNA4 is a ligand-gated ion channel that mediates fast signal transmission at synapses. Several genetic studies have focused on the *CHRNA4* gene as a candidate gene for ADHD due to its involvement in the nicotinic acetylcholine system; however, they have conflicting results. Comings et al. found a dinucleotide repeat in intron 1 as a risk for ADHD, Todd et al. identified polymorphism in the exon 2–intron 2 junction as a risk for severe inattention problems, Brookes et al. found the association between the 5’ flanking regions of *CHRNA4* with combined subtype of ADHD; nevertheless, Kent et al. and Bobb et al. found no significant evidence of association in their studies (Kent et al., 2001b; Todd et al., 2003; Bobb et al., 2005; Brookes et al., 2006; Lee et al., 2008; Wallis et al., 2009).

Cholinergic receptor nicotinic alpha 7 subunits (*CHRNA7*) is a gene coding for the a7 subunit of the neuronal acetylcholine receptor (CHRNA7). CHRNA7 is also ligand-gated ion channels that mediate fast signal transmission at synapses and is found in both presynaptic and glial cell membranes. It is more prominent in the hippocampus and GABAergic interneurons of stratum oriens, stratum radiatum, and on pyramidal neurons (Bates et al., 2014). It facilitates the transmission of GABA from the synapse to the postsynaptic membrane and glial cells. The study by Kent et al. (2001a) showed that there was no association between CHRNA7 microsatellite markers and ADHD. Deletion at 15q13 spanning *KLF13* and *CHRNA7* has been reported to relate to ADHD (Valbonesi et al., 2015). Although this CNV spans two genes, *CHRNA7* is mostly likely the cause as *CHRNA7* duplications at 15q13.3 has also been linked with ADHD (Gillentine et al., 2017). There are few association studies representing *CHRNA4* and *CHRNA7* genes as candidate genes for ADHD. Further analyses should be done to clarify the possible effects of the cholinergic neurotransmitter system on the pathogenesis of childhood ADHD.

##### Genes and copy number variations related to glutamatergic and GABAergic pathway

Glutamate is the major excitatory neurotransmitter in the brain and is involved in multiple functions relevant to ADHD: brain development, modulation of neuronal activity, regulation of dopamine signaling, synaptic plasticity, memory formation, and learning. GABA is an inhibitory neurotransmitter in the brain which is produced from the conversion of glutamate *via* glutamic acid decarboxylase (GAD) enzyme. Glutamate plays a role in ADHD through bidirectional regulation of dopamine signaling while GABA modulates dopamine metabolism. A study done based on proton magnetic resonance spectroscopy revealed higher levels of glutamate and low levels of GABA in children with ADHD than in controls (Courvoisie et al., 2004). This imbalance leads to symptoms of ADHD such as inattention, hyperactivity, and impulsivity hence showing the biological basis for ADHD. Reported genes include *GAD1*, glutamate receptor genes (*GRM7, GRM5, GRM8*, and *GRM1*). *GAD1* (Glutamate decarboxylase 1) encodes a key enzyme of GABA biosynthesis (GAD67). GAD67 catalyzes the conversion of glutamic acid to gamma-aminobutyric acid (GABA) and is found in the presynaptic membrane and highly expressed in the frontal cortex. Bruxel et al. (2016) performed the first study that showed a positive association between the *GAD1* gene and ADHD.

Glutamate receptor metabotropic (GRMs) are GPCRs involved in the modulation of excitatory synaptic transmission (Taniura et al., 2006). There are three receptor groups that are based on putative signal transduction mechanisms, sequence homology, and pharmacologic properties (Conn and Pin, 1997). GRM1 and GRM5 are members of group 1 and are expressed in the basal ganglia and cerebellum (Berthele et al., 1999). *GRM1* knockout mice demonstrated the involvement of this receptor in associative learning due to reduced hippocampal long-term potentiation (Aiba et al., 1994a; Gil-Sanz et al., 2008) as well as in motor learning due to deficient cerebellar long-term depression (Aiba et al., 1994b). Impaired GRM5 receptor function results in inappropriate retention of aversive memories leading to anxiety disorders; therefore, it seems to be critical for inhibitory learning mechanisms (Xu et al., 2009). GRM7 and GRM8 are members of group 3 that inhibit the cyclic AMP cascade. GRM7 is an important presynaptic regulator of neurotransmission in the mammalian central nervous system. It has been linked to anxiety (Cryan et al., 2003) and is the most highly conserved GRM member across multiple species (Makoff et al., 1996). *GRM4* rs1906953 and *GRM7* rs9826579 are associated with ADHD in the Chinese population (Zhang et al., 2021b). *GRM7* rs37952452 polymorphism was reported to play a role in the treatment response to methylphenidate in children with ADHD (Park S. et al., 2013), and it was replicated in another study (Park et al., 2014). *GRID2* (Glutamate Ionotropic Receptor Delta Type Subunit 2) increases the risk of ADHD too (Zhang et al., 2021c). The *GRM8* null mutant mice showed novelty-induced hyperactivity and altered fear responses (Gerlai et al., 2002; Fendt et al., 2010). Anxiety disorders, motor coordination problems, and learning disorders are common features found in ADHD cases (Rommelse et al., 2009).

Approximately 10% of the children with ADHD carried CNVs in the glutamate metabotropic genes (*GRM5, GRM7, GRM8*, and *GRM1*) or in genes known to interact with them (Elia et al., 2011). A high rate of copy number variations in children with ADHD compared to controls has been reported (Williams et al., 2010) and replicated (Yang et al., 2013). Most of the identified CNVs in children with ADHD carry glutamate metabotropic genes (Elia et al., 2011; Liu et al., 2021b).

##### Glutamatergic and GABAergic pathway and environmental interactions

Manganese is an important cofactor in the synthesis of anti-oxidative enzymes (Bresciani et al., 2015) and is an essential metal that plays a fundamental role in brain development and functioning. Hence, its deficiency can lead to oxidative stress. Although an excessive amount of manganese can also damage the nerves and disturb the glutamine-glutamate-GABAergic cycle which is vital for proper brain functioning, especially in the frontal cortex (Sidoryk-Wegrzynowicz, 2014). Manganese hair levels have been associated with ADHD in children (Collipp et al., 1983). Moreover, developmental exposure to manganese in laboratory animals led to hyperactivity (Banerjee et al., 2007).

#### Genes and copy number variations related to other pathways

TAR RNA Binding Protein 1 (*TARBP1)* DNA methylation is related to ADHD symptoms in adulthood and childhood (Weiß et al., 2021). *ADGRL3*-rs1565902 is linked with ADHD in the Caribbean population (Cervantes-Henriquez et al., 2021). Atrophilin-3 [LPHN3; or ADGRL (3)] is a synaptic adhesion G protein-coupled receptor that binds to fibronectin leucine rich transmembrane protein 3 and teneurin-3 (FLRT3 and TEN-3) (Regan et al., 2019). The rs2122642-*ADGRL3* (C allele) and *ADGRL3* haplotype CCC (markers rs1565902-rs10001410-rs2122642) are associated with ADHD in the Caribbean community (Puentes-Rozo et al., 2019). Mice null for Lphn3 exhibit high levels of dopamine and serotonin in the dorsal striatum and present with a hyperactive phenotype suggesting its role in the modulation of monoamine signaling (Wallis et al., 2012). A null mutation of Lphn3 (KO) in Sprague-Dawley rats was related to hyperactivity and striatal changes in dopamine markers (Regan et al., 2019). Fibroblast Growth Factor 1 (FGF1) -rs2282794 has been reported to relate to ADHD among the Caribbean population too (Cervantes-Henriquez et al., 2021). Monoamine Oxidase A (MAO) is encoded by the *MAOA* gene and is highly expressed in the frontal cortex, responsible for the oxidation of neurotransmitters and dietary amines including serotonin, norepinephrine, and dopamine. Das et al. (2006) identified that the short 3.5 repeat allele of the MAOA-u VNTR, is associated with ADHD in children. Moreover, the 6-repeat allele of the CA microsatellite and the G-allele of the 941G/T SNP of MAOA have been reported (Domschke et al., 2005). Brain-derived neurotrophic factor (BDNF) is important for the development of GABAergic neurons (Yamada et al., 2002; Hong et al., 2008). Patients with ADHD have been reported to have *BDNF* mutations (Kwon et al., 2015). Syntaxin1, SNAP25, and VAMP2 form a SNARE complex that is responsible for the regulation of neurotransmitter release (Rizo and Südhof, 2012). *SNAP25* and *STX1A* polymorphisms have been reported to associate with ADHD (Gao et al., 2015).

Other acknowledged CNVs include deletions at 2p16.3 spanning *NRXN1*, 15q11.2, 15q13.3 spanning *BP4* and *BP4.5-BP5* and 22q11.21, and duplications at 1q21, 16p11.2, 16p13.11, and 22q11.21 (Gudmundsson et al., 2019). Deletions and duplications at the 15q13 and 16p11.2 and deletion and duplication of *PARK2* locus at 6q25.2-q27 (Jarick et al., 2014). Additionally, 16p13.11 and 15q13.3, 3p26 deletion or duplication spanning the *CNTN6* gene have been reported (Williams et al., 2010, 2012; Jarick et al., 2014; Hu et al., 2015; Zarrei et al., 2019). *De novo* aberrations at four CNVs: 15q13.1-13.2 duplication, 16p13.11 duplication, 16p12.2 deletion, and 22q11.21 duplication have been implicated in ADHD studies too (Martin et al., 2020).

#### Other microelement dysfunction related to unknown pathways

Nutritional deficiencies have been increasingly implicated as possible risk factors for ADHD (Sinn and Bryan, 2007; Verlaet et al., 2014). Low levels of copper, iron, zinc, magnesium, and omega-3 fatty acids have been detected in children with this disorder while sugar, artificial food colorants, and preservatives have been associated with an increased risk of ADHD (Holtkamp et al., 2004; Cormier and Elder, 2007; Mahmoud et al., 2011; San Mauro Martín et al., 2018). Iron, zinc, and magnesium supplements act as novel therapies for certain aspects of ADHD (Konofal et al., 2008).

Hair mercury and manganese are considered to be the other developmental toxicants related to the development of ADHD symptomatology (Banerjee et al., 2007; Boucher et al., 2012). Mercury is a potent neurodevelopmental toxicant commonly encountered in the environment in dietary or non-dietary form. Small prenatal exposures resulting from maternal consumption of contaminated fish adversely affected intelligence, language development, gross motor skills, visual-spatial skills, memory, and attention in offspring (Anderson et al., 1981; Collipp et al., 1983). Duration and dosage of mercury exposure determine the incidence and prevalence of ADHD according to one meta-analysis (Yoshimasu et al., 2014).

Lead is known as an environmental toxin that negatively affects brain development (Foltinová et al., 2007). Low levels of lead exposure can result to inattention, low intelligence, and behavioral problems (Lanphear et al., 2005; Nevin et al., 2008). High serum lead levels have an association with ADHD (Boucher et al., 2012). A recent meta-analysis of children and adolescents demonstrated a significant association between lead exposure and symptoms of both inattention and hyperactivity/impulsivity (Goodlad et al., 2013).

#### Treatments

##### Pharmacological treatments

The treatment options include pharmacological and non-pharmacological. Pharmacological treatments are helpful as they restore the balance of the neurotransmitters in the prefrontal cortex (Seixas et al., 2012; Burcu et al., 2016). Commonly used drugs include stimulants such as methylphenidate and amphetamines as well as non-stimulants such as atomoxetine and α-2 agonists (guanfacine and clonidine) (Reddy, 2013; Sharma and Couture, 2014; Tarver et al., 2014; Catalá-López et al., 2015). These drugs have many side effects ranging from mild to severe ones (Tobaiqy et al., 2011; Clavenna and Bonati, 2017). Despite the availability of pharmacotherapy, the outcomes differ according to the individuals; about 80% of the patients respond well to the psychostimulant drugs while approximately 20% have a poor response (Kendall et al., 2008; Wolraich et al., 2019). Therefore, there is a need to explore and develop more efficacious drugs with less side effects.

##### Dietary treatments

Low levels of copper, iron, zinc, magnesium, and omega-3 fatty acids have been detected in children with this disorder while sugar, artificial food colorants, and preservatives have been associated with an increased risk of ADHD (Holtkamp et al., 2004; Konofal et al., 2004; Cormier and Elder, 2007; Lahat et al., 2011; San Mauro Martín et al., 2018). Iron, zinc, and magnesium supplements act as novel therapies for certain aspects of ADHD (Konofal et al., 2008). Low levels of omega-3 fatty acids and the utilization of sugar, artificial food colorants, and preservatives have been reported to associate with an increased risk of ADHD (Millichap and Yee, 2012). Several dietary treatments have been suggested including sugar-restricted, additive, and salicylate-free (Feingold diet), oligoantigenic, ketogenic, megavitamin, and polyunsaturated fatty acid supplements (PUFA) (Howard et al., 2011; Millichap and Yee, 2012). Nevertheless, the role of diet and dietary supplements in the cause and treatment of ADHD in children is still controversial (Millichap and Yee, 2012).

## Conclusion

By using a systematic search that combines results from different studies, we provide evidence that the etiology of ADHD in children is multifactorial. Genetic and environmental/social factors have been reported to play a role by altering neurotransmission. SNPs, copy number variations, and microRNAs have been linked with ADHD. The imbalance of the neurotransmitters leads to ADHD. *DRD1, DRD2, DRD4, DAT1, DBH, NET1/SLC6A2, ADRA2C, ADRA2A, CHRNA4, CHRNA7*, and *MAO* is involved in dopaminergic pathways directly or indirectly. *TPH2, HTR1A, HTR1B*, and *HTR2A* are involved in the serotonergic pathway. *SNAP-25* and *BDNF* are involved in neurotransmission and neuronal plasticity. *GAD1* is involved in the GABAergic pathway. SNPs that fall under the dopaminergic and serotonergic pathways appear to contribute more to the possible etiology. Dysregulation/exposure to environmental factors including nicotine, ethanol, copper, iron, zinc, magnesium, lead, and manganese can also interfere with neurotransmission. The reported SNPs differ according to ethnic groups. MicroRNAs are responsible for post-transcription modulation of the genes, and studies have shown their possible roles in the regulation of some of the ADHD-related genes. For instance, miR22-3p can regulate *BDNF, HTR2C*, and *MAO*, miR-138-1 and miR-296 can regulate *BDNF*, miR-96 can regulate *HTR1B*, miR-30b-5p, miR-6070, miR-1301, and miR-384-5p can regulate *DAT1* (Supplementary Table 2). Although there are some animal model studies, most of them are KO and do not generate the genetic alteration of the patients. The majority of the available animal models are those related to the dopaminergic pathway. Epigenetic changes such as SUMOylation, methylation, and acetylation have been reported in genes related to the dopaminergic pathway.

Consequently, the dopaminergic pathway remains to be crucial in the pathogenesis of ADHD. It can be affected by environmental factors and other proposed pathways including adrenergic, GABAergic, glutamatergic, serotonergic, and cholinergic, directly or indirectly. At least one of the five dopaminergic receptors is located in adrenergic, GABAergic, glutamatergic, and cholinergic neurons: DRD1, DRD2, dopamine D3 receptor (DRD3), DRD4, and dopamine D5 receptor (DRD5) (Robertson and Staines, 1994; Hersch et al., 1995; Centonze et al., 2002, 2003; Rivera et al., 2002, 2003; Berlanga et al., 2005; Mizuno et al., 2007; Xi and Gardner, 2007). Animal models of ADHD showed dysregulation of dopamine functions and abnormal behaviors of which were normalized upon administration of stimulant medications (Russell, 2011). Some studies based on children with ADHD identified frontal cortical regions, which are rich in dopamine, as sites related to ADHD (McAlonan et al., 2007, 2009). Additionally, several studies have shown that the dopamine receptor density in several brain regions of ADHD patients is lower compared to healthy individuals (Jönsson et al., 1999; Medin et al., 2013). Methylphenidate is the first-line stimulant drug for ADHD. It improves the symptoms of ADHD in about 70% of the patients (Hodgkins et al., 2012; Bolea-Alamañac et al., 2014). It acts through several pathways including dopaminergic, noradrenergic, serotonergic, glutamatergic, and GABAergic to increase the levels of dopamine in synaptic cleft as it was reported by recent studies on human and animal models (Millan et al., 2000; Oades, 2008; Wilens, 2008; Freese et al., 2012; Park et al., 2014). Preclinical studies, as well as the dissection of the mechanism of action of available stimulant drugs, support the view that dopaminergic, serotonergic, glutamatergic and GABAergic systems are functionally interconnected. These observations open the field for the exploration of the physical interconnections between receptors and neurotransmitters. Table 1 summarizes different neurotransmitters and neuroreceptors and the roles they play in the pathophysiology and pathogenesis of ADHD.

### The limitations and strengths of this review

This is a comprehensive review covering genetics, neurotransmitters as well as neuroanatomical structures involved in ADHD. Nevertheless, ADHD being a heterogeneous illness with a varying number of comorbidities, it is still unclear how environmental factors relate to all neurotransmitter pathways. Future studies should explore more the associations between environmental factors and neurotransmitter pathways. Although several genes have been related to ADHD, there are few animal model studies on the majority of the genes. More epigenetic studies are required for other genes on top of those related to the dopaminergic pathway.

## Author contributions

MK, HD, and JX performed a literature review, drafted the article, and gave final approval of the version to be published. BC and YM assisted in the preparation of the table, revised the manuscript, and gave final approval of the version to be published. LY, FH, OB, and JP revised the manuscript critically for important intellectual content and gave final approval of the version to be published. FY conceptualized, designed, and revised the manuscript critically for important intellectual content and gave final approval of the version to be published. All authors contributed to the article and approved the submitted version.
