# Supplementary material for: Attention-deficit/hyperactive disorder updates
Source: Front Mol Neurosci. 2022 Sep 21;15:925049. doi: 10.3389/fnmol.2022.925049 (PMC9532551; doi:10.3389/fnmol.2022.925049)
Supplement: Supplementary file 1 [file Data_Sheet_1.doc]

**Supplementary Table 1 Genetic biomarkers of the attention-deficit/hyperactive disorder**

| **Gene** | **Gene name** | **Pathway/Function** | **Location** | **Risk Allele** | **Polymorphism** | **Population/Study area** | **Reference (s)** |
| --- | --- | --- | --- | --- | --- | --- | --- |
| *DRD1* | Dopamine receptor D1 | Dopaminergic pathway | Chr5:174863257 | C allele | rs265977 | Caucasian | (Ribasés et al., 2012) |
|  |  |  | Chr5:174870902 | T allele | rs265981 | Toronto | (Luca et al., 2007) |
|  |  |  | Chr5:174870150 | C allele | rs4532 |  |  |
|  |  |  | Chr5:174868700 | G allele | rs686 |  |  |
| *DRD2* | Dopamine receptor D2 | Dopaminergic pathway | Chr11:113270828 | Taq1A/ T allele | rs1800497 | Finland | (Neville et al., 2004; Pan et al., 2015; Kim et al., 2018) |
|  |  |  | Chr11:113300022 | A allele | rs7131465 | Italy | (Mariggiò et al., 2021) |
|  |  |  | Chr11:113270828 | G allele | rs1800497 | Finland | (Nyman et al., 2007) |
|  |  |  | Chr11:113289182 | T allele | rs1079727 |  |  |
|  |  |  | Chr11:113282090 | G allele | rs1124491 |  |  |
|  |  |  | Chr11:113282669 | A allele | rs1079595 |  |  |
|  |  |  | 3′-UTR | Unknown | Taq1A RFLP |  |  |
| *DRD4* | Dopamine DR receptor 4 | Dopaminergic pathway | Unknown | 7-repeat allele | VNTR | USA | (Bidwell et al., 2011) |
|  |  |  | Exon 3 | 7-repeat allele | VNTR | UK | (Brookes et al., 2006) |
|  |  |  | Exon 3 | 7-repeat allele | VNTR | USA | (Faraone et al., 1999) |
|  |  |  | Promoter region | allele 2 | 120 bp duplication | UK | (Mill et al., 2003) |
|  |  |  | Promoter region | C allele | 616 C/G substitution |  |  |
|  |  |  | Promoter region | C allele | 521 C/T substitution |  |  |
|  |  |  | Exon 3 | 4R | VNTR | Egypt | (Shahin et al., 2015) |
|  |  |  | Chr11:637014 | C allele | rs916457 | Colombia | (Cervantes-Henriquez et al., 2021) |
| *DAT1* | Dopamine Transporter 1 | Dopaminergic pathway | Chr5:1412645 and Chr5:1409127 | 3'UTR, 10-repeat allele | Haplotype rs27048 (C)/rs429699 (T) | Taiwan | (Shang et al., 2011) |
|  |  |  | Chr5:1411412 | A-allele | rs6347 | UK | (Brookes et al., 2006) |
|  |  |  | Unknown | Intron 8, 10-repeat allele and 3-repeat allele | VNTR |  |  |
|  |  |  | Chr5:1394815 and chr5:1407104 | Intron 10 and intron 13 | rs1042098 and rs3776513 |  |  |
|  |  |  | Chr5:1446389,unknown, Chr5:1450413 | 5' flanking region | rs2652511, rs10070282 and rs2550946 |  |  |
|  |  |  | Unknown | 3'-UTR, 10-repeat allele | VNTR | Taiwan | (Chen et al., 2003) |
|  |  |  | Chr5:1394272 | 3'-UTR, 10-repeat allele | rs27072 | Canada | (Ouellet-Morin et al., 2008) |
| *TPH2* | Tryptophan hydroxylase 2 | Serotonergic pathway | Chr12:72377312 | G allele | rs11179027 | Korea | (Park et al., 2013b) |
|  |  |  | Chr12:72348698 | G allele | rs1843809 |  |  |
|  |  |  | Chr12:72348698 | T allele | rs1843809 | UK | (Brookes et al., 2006) |
|  |  |  | Chr12:72355179 | A allele | rs1386493 |  |  |
|  |  |  | Chr12:72388374 | G allele | rs1007023 |  |  |
|  |  |  | Chr12:72331923 | G allele | rs4570625 | Germany | (Walitza et al., 2005) |
|  |  |  | Chr12:72332153 | T allele | rs11178997 |  |  |
| *HTR1A* | 5-hydroxytryptamine receptor 1A | Serotonergic pathway | Chr5:63257292 | C allele | Unknown | Korea | (Shim et al., 2010) |
| *HTR2A* | 5-hydroxytryptamine receptor 2A | Serotonergic pathway | Chr13:47429446 | T allele | rs7984966 | UK | (Pinto et al., 2016) |
|  |  |  | Chr13:47410103 | T allele | rs7322347 |  |  |
| *HTR1B* | 5-hydroxytryptamine receptor 1B | Serotonergic pathway | Chr6:78172260, Chr6:78171941, Chr6:78172750, Chr6:78172992, Chr6:78173281, Chr6:78173382 | C allele, C allele, A allele, G allele, T allele, A allele | rs6296, rs6297, rs130060, rs6298, rs130058 and rs11568817 | USA | (Levitan et al., 2002) |
| *SLC6A4* | Solute Carrier Family 6 Member 4 | Serotonergic pathway | 5HTTLPR 44-bp insertion/deletion | Not applicable | Not applicable | Several countries | (Gizer et al., 2009) |
| *DBH* | Dopamine beta-hydroxylase | Dopaminergic pathway | Chr9:136500515 | T allele | rs1611115 | India | (Bhaduri et al., 2010) |
|  |  |  | Chr9:136505114 | A allele | rs1108580 |  |  |
|  |  |  | Chr9:136509634 | T allele | rs2519152 |  |  |
|  |  |  | Chr9:136500515 | C allele | rs1611115 | Brazil | (Kieling et al., 2008) |
|  |  |  | Chr9:136522274 | C allele | rs6271 | USA | (Tang et al., 2006) |
|  |  |  | Chr9:136509634 | Taq1A | rs2519152 |  |  |
| *NET1/SLC6A2* | Neuroepithelial cell transforming gene 1 | Dopaminergic pathway | Unknown | C allele | rs3785143 | USA | (Biederman et al., 2008) |
| *ADRA2C* | Alpha-2C-adrenergic receptor | Dopaminergic pathway | Chr4:3765225 | T allele | rs7682295 | China | (Guan et al., 2009) |
| *ADRA2A* | Alpha-2A-adrenergic receptor | Dopaminergic pathway | Chr10:112839579 | A allele | rs553668 | China | (Wang et al., 2021). |
| *CHRNA4* | Cholinergic Receptor nicotinic alpha 4 subunit | Dopaminergic pathway | Unknown | 5′ flanking region | Unknown | UK | (Brookes et al., 2006) |
| *KLF13 and CHRNA7* | Kruppel like factor 13 and Cholinergic Receptor nicotinic alpha 7 subunit respectively | Immune/inflammatory and oxidative stress signaling pathways and dopamine respectively | 15q13 deletion | Not applicable | Not applicable | USA | (Valbonesi et al., 2015) |
| *GRM4* | Glutamate receptor metabotropic 4 | Modulation of excitatory synaptic transmission | Chr6:34036446 | C allele | rs1906953 | China | (Zhang et al., 2021) |
| *GRM7* | Glutamate receptor metabotropic 7 | Modulation of excitatory synaptic transmission | Chr3:7782371 | T allele | rs9826579 | China | (Zhang et al., 2021) |
|  |  |  | Chr3:7666784 | C allele | rs3792452 | Korea | (Park et al., 2013a, 2014) |
| *GAD1* | Glutamate decarboxylase 1 | GABAergic pathway | Chr2:171673375 | G allele | rs3749034 | Brazil | (Bruxel et al., 2016) |
|  |  |  | Unknown | C allele | rs11542313 |  |  |
| *ADGRL3*/ *LPHN3* | Adhesion G Protein-Coupled Receptor L3) | Cell-cell adhesion and neuron guidance | Chr4:62408620 | T allele | rs1565902 | Caribbean | (Cervantes-Henriquez et al., 2021) |
|  |  |  | Chr4:62698264 | C allele | rs2122642 |  | (Puentes-Rozo et al., 2019) |
| *FGF1* | **Fibroblast Growth Factor 1** |  | Chr5:141981709 | G allele | rs2282794 | Caribbean | (Cervantes-Henriquez et al., 2021) |
| *MAO* | Monoamine Oxidase A | Oxidation of neurotransmitters | Unknown | VNTR | Unknown | Indian | (Das et al., 2006) |
|  |  |  | Unknown | 941G/T SNP | Unknown | Ireland | (Domschke et al., 2005) |
| *BDNF* | Brain-derived neurotrophic factor | Regulator of the synaptic transmission and plasticity | Chr11:27680744 | A allele | rs11030101 | Korean | (Kwon et al., 2015) |
|  |  |  | Chr11:27695910 | C allele | rs10835210 |  |  |
| *SNAP25* | **Synaptosomal-associated protein 25 kDa** | N-ethylmaleimide-sensitive attachment protein receptor | Chr20:10220496 | G allele | rs363039 | China | (Gao et al., 2015). |
| *STX1A* | **Syntaxin 1A** | N-ethylmaleimide-sensitive attachment protein receptor | Chr7:73127098 | G allele | rs875342 | China | (Gao et al., 2015). |

**Supplementary Table 2 MicroRNAs related to the attention-deficit/hyperactive disorder both in human and animal models**

| **MicroRNA** | **Gene regulated/Function** | **Status in ADHD** | **Population/Study area for the animal models** | **Reference (s)** |
| --- | --- | --- | --- | --- |
| miR18a-5p | Unknown | Decreased | Turkey | (Kandemir et al., 2014) |
| miR22-3p | *BDNF, HTR2C, MAOA*, and *RGS2* | Decreased | Turkey | (Kandemir et al., 2014) |
| miR24-3p | Oxidative stress | Decreased | Turkey | (Kandemir et al., 2014) |
| miR106b-5p | Oxidative stress | Decreased | Turkey | (Kandemir et al., 2014) |
| miR125b-5p | Unknown | Decreased | Turkey | (Kandemir et al., 2014) |
| miR107 | Unknown | Decreased | Turkey | (Kandemir et al., 2014) |
| miR155a-5p | Unknown | Increased | Turkey | (Kandemir et al., 2014) |
| miRNA let-7d | *LGALS3* | Increased | China | (Wu et al., 2015) |
| miR-6070 | *DAT1 (SLC6A3)* | Not applicable | Czech Republic | (Šerý et al., 2015) |
| miR-138-1 | *Nr3c1* and *Bhlhb2* for the regulation of the *BDNF* | Decreased | China | (Wu et al., 2017) |
| miR-296 | *Nr3c1* and *Bhlhb2* for the regulation of the *BDNF* | Decreased | China | (Wu et al., 2017) |
| miR-34c | *Nr3c1* and *Bhlhb2* | Decreased | China | (Wu et al., 2017) |
| miR-494 | *Nr3c1* and *Bhlhb2* | Decreased | China | (Wu et al., 2017) |
| miR-34c-3p | ET, HMGA2 and NOTCH2 | Increased | Spain | (Garcia-Martínez et al., 2016) |
| miR-96 | *HTR1B* | Not applicable | Spain | (Sánchez-Mora et al., 2013) |
| miR-641 | *SNAP-25* | Not applicable | Hungary | (Németh et al., 2013) |
| miR-30b-5p | *DAT1 (SLC6A3)* | Not applicable | Czech Republic | (Šerý et al., 2015) |
| miR-1301 | *DAT1 (SLC6A3)* | Not applicable | Czech Republic | (Šerý et al., 2015) |
| miR-384-5p | *DAT* and *CREB* | Not applicable | China. | (Xu et al., 2019) |
| miR-132-3p | Unknown | Increased | Turkey | (Coskun et al., 2021) |
| miR-5692b | Unknown | Increased | Turkey | (Aydin et al., 2019) |

**Supplementary Table 3 Environmental/biochemical biomarkers for ADHD**

| **Risk exposure** | **Biomarker** | **Origin of biomarker** | **Status** | **Function/Pathway/Effect** | **Population/Study area** | **Author and years** |
| --- | --- | --- | --- | --- | --- | --- |
| Manganese | Manganese | Blood | Elevated | Neurotoxic -dopamine neurotransmission | Korea | (Hong et al., 2018) |
| Trichlorophenols | Trichlorophenols | Urine | Increased | Neurodevelopmental toxicants | USA | (Xu et al., 2019) |
| Maternal alcohol | Ethyl glucuronide | Meconium | Increased | Neurodevelopmental toxicants | Germany | (Eichler et al., 2018) |
| Organophosphate pesticide | Dialkylphosphate metabolites | Urine | Increased | Decreases in brain cholinergic receptors | Taiwan | (Yu et al., 2016) |
| Prenatal exposure to methylmercury | Methylmercury | Blood | Increased |  | Canada | (Boucher et al., 2012) |
| Lead | Lead | Blood | Increased | Dopamine system | UAE. | (Boucher et al., 2012) |
| Smoking | Cotinine | Urine | Increased | Adrenergic pathway | Korea | (Cho et al., 2013) |
| Pyrethroid pesticides | 3-phenoxybenzoic acid | Urine | Increased | Dopamine system | Korea | (Lee et al., 2020) |
| Iron deficiency | Ferritin levels | Serum | Decreased | Production of neurotransmitters such as dopamine, nor-epinephrine and serotonin | Israel and Egypt | (Konofal et al., 2004; Lahat et al., 2011) |
| Zinc | Zinc | Serum | Decreased | Antioxidant | Egypt | (Mahmoud et al., 2011) |
| Magnesium | Magnesium | Serum | Decreased | Protects cell membranes from excitatory neurotransmitters such as glutamate | Egypt | (Mahmoud et al., 2011) |
| Norepinephrine, epinephrine | Normetanephrine and vanillylmandelic acid. | Urine | Increased | Noradrenergic or adrenergic | Texas | (Pliszka et al., 1994) |

**References**

Aydin, S. U., Kabukcu Basay, B., Cetin, G. O., Gungor Aydin, A., and Tepeli, E. (2019). Altered microRNA 5692b and microRNA let-7d expression levels in children and adolescents with attention deficit hyperactivity disorder. *J. Psychiatr. Res.* 115, 158–164. doi:10.1016/j.jpsychires.2019.05.021.

Bhaduri, N., Sarkar, K., Sinha, S., Chattopadhyay, A., and Mukhopadhyay, K. (2010). Study on DBH genetic polymorphisms and plasma activity in attention deficit hyperactivity disorder patients from Eastern India. *Cell. Mol. Neurobiol.* 30, 265–274. doi:10.1007/s10571-009-9448-5.

Bidwell, L. C., Willcutt, E. G., McQueen, M. B., DeFries, J. C., Olson, R. K., Smith, S. D., et al. (2011). A family based association study of DRD4, DAT1, and 5HTT and continuous traits of attention-deficit hyperactivity disorder. *Behav. Genet.* 41, 165–174. doi:10.1007/s10519-010-9437-y.

Biederman, J., Kim, J. W., Doyle, A. E., Mick, E., Fagerness, J., Smoller, J. W., et al. (2008). Sexually dimorphic effects of four genes (COMT, SLC6A2, MAOA, SLC6A4) in genetic associations of ADHD: a preliminary study. *Am. J. Med. Genet. Part B, Neuropsychiatr. Genet. Off. Publ. Int. Soc. Psychiatr. Genet.* 147B, 1511–1518. doi:10.1002/ajmg.b.30874.

Boucher, O., Jacobson, S. W., Plusquellec, P., Dewailly, E., Ayotte, P., Forget-Dubois, N., et al. (2012). Prenatal methylmercury, postnatal lead exposure, and evidence of attention deficit/hyperactivity disorder among Inuit children in Arctic Québec. *Environ. Health Perspect.* 120, 1456–1461. doi:10.1289/ehp.1204976.

Brookes, K., Xu, X., Chen, W., Zhou, K., Neale, B., Lowe, N., et al. (2006). The analysis of 51 genes in DSM-IV combined type attention deficit hyperactivity disorder: association signals in DRD4, DAT1 and 16 other genes. *Mol. Psychiatry* 11, 934–953. doi:10.1038/sj.mp.4001869.

Bruxel, E. M., Akutagava-Martins, G. C., Salatino-Oliveira, A., Genro, J. P., Zeni, C. P., Polanczyk, G. V, et al. (2016). GAD1 gene polymorphisms are associated with hyperactivity in Attention-Deficit/Hyperactivity Disorder. *Am. J. Med. Genet. Part B, Neuropsychiatr. Genet. Off. Publ. Int. Soc. Psychiatr. Genet.* 171, 1099–1104. doi:10.1002/ajmg.b.32489.

Cervantes-Henriquez, M. L., Acosta-López, J. E., Ahmad, M., Sánchez-Rojas, M., Jiménez-Figueroa, G., Pineda-Alhucema, W., et al. (2021). ADGRL3, FGF1 and DRD4: Linkage and Association with Working Memory and Perceptual Organization Candidate Endophenotypes in ADHD. *Brain Sci.* 11. doi:10.3390/brainsci11070854.

Chen, C.-K., Chen, S.-L., Mill, J., Huang, Y.-S., Lin, S.-K., Curran, S., et al. (2003). The dopamine transporter gene is associated with attention deficit hyperactivity disorder in a Taiwanese sample. *Mol. Psychiatry* 8, 393–396. doi:10.1038/sj.mp.4001238.

Cho, S. C., Hong, Y. C., Kim, J. W., Park, S., Park, M. H., Hur, J., et al. (2013). Association between urine cotinine levels, continuous performance test variables, and attention deficit hyperactivity disorder and learning disability symptoms in school-aged children. *Psychol. Med.* 43, 209–219. doi:10.1017/S0033291712001109.

Coskun, S., Karadag, M., Gokcen, C., and Oztuzcu, S. (2021). miR-132 and miR-942 Expression Levels in Children with Attention Deficit and Hyperactivity Disorder: A Controlled Study. *Clin. Psychopharmacol. Neurosci. Off. Sci. J. Korean Coll. Neuropsychopharmacol.* 19, 262–268. doi:10.9758/cpn.2021.19.2.262.

Das, M., Bhowmik, A. Das, Sinha, S., Chattopadhyay, A., Chaudhuri, K., Singh, M., et al. (2006). MAOA promoter polymorphism and attention deficit hyperactivity disorder (ADHD) in indian children. *Am. J. Med. Genet. Part B, Neuropsychiatr. Genet. Off. Publ. Int. Soc. Psychiatr. Genet.* 141B, 637–642. doi:10.1002/ajmg.b.30385.

Domschke, K., Sheehan, K., Lowe, N., Kirley, A., Mullins, C., O’sullivan, R., et al. (2005). Association analysis of the monoamine oxidase A and B genes with attention deficit hyperactivity disorder (ADHD) in an Irish sample: preferential transmission of the MAO-A 941G allele to affected children. *Am. J. Med. Genet. Part B, Neuropsychiatr. Genet. Off. Publ. Int. Soc. Psychiatr. Genet.* 134B, 110–114. doi:10.1002/ajmg.b.30158.

Eichler, A., Hudler, L., Grunitz, J., Grimm, J., Raabe, E., Goecke, T. W., et al. (2018). Effects of prenatal alcohol consumption on cognitive development and ADHD-related behaviour in primary-school age: a multilevel study based on meconium ethyl glucuronide. *J. Child Psychol. Psychiatry.* 59, 110–118. doi:10.1111/jcpp.12794.

Faraone, S. V, Biederman, J., Weiffenbach, B., Keith, T., Chu, M. P., Weaver, A., et al. (1999). Dopamine D4 gene 7-repeat allele and attention deficit hyperactivity disorder. *Am. J. Psychiatry* 156, 768–770. doi:10.1176/ajp.156.5.768.

Gao, Q., Liu, L., Chen, Y., Li, H., Yang, L., Wang, Y., et al. (2015). Synaptosome-related (SNARE) genes and their interactions contribute to the susceptibility and working memory of attention-deficit/hyperactivity disorder in males. *Prog. Neuropsychopharmacol. Biol. Psychiatry* 57, 132–139. doi:10.1016/j.pnpbp.2014.11.001.

Garcia-Martínez, I., Sánchez-Mora, C., Pagerols, M., Richarte, V., Corrales, M., Fadeuilhe, C., et al. (2016). Preliminary evidence for association of genetic variants in pri-miR-34b/c and abnormal miR-34c expression with attention deficit and hyperactivity disorder. *Transl. Psychiatry* 6, e879. doi:10.1038/tp.2016.151.

Gizer, I. R., Ficks, C., and Waldman, I. D. (2009). Candidate gene studies of ADHD: a meta-analytic review. *Hum. Genet.* 126, 51–90. doi:10.1007/s00439-009-0694-x.

Guan, L., Wang, B., Chen, Y., Yang, L., Li, J., Qian, Q., et al. (2009). A high-density single-nucleotide polymorphism screen of 23 candidate genes in attention deficit hyperactivity disorder: suggesting multiple susceptibility genes among Chinese Han population. *Mol. Psychiatry* 14, 546–554. doi:10.1038/sj.mp.4002139.

Hong, J. H., Hwang, I. W., Lim, M. H., Kwon, H. J., and Jin, H. J. (2018). Genetic associations between ADHD and dopaminergic genes (DAT1 and DRD4) VNTRs in Korean children. *Genes Genomics* 40, 1309–1317. doi:10.1007/s13258-018-0726-9.

Kandemir, H., Erdal, M. E., Selek, S., Ay, Ö. İ., Karababa, I. F., Kandemir, S. B., et al. (2014). Evaluation of several micro RNA (miRNA) levels in children and adolescents with attention deficit hyperactivity disorder. *Neurosci. Lett.* 580, 158–162. doi:10.1016/j.neulet.2014.07.060.

Kieling, C., Genro, J. P., Hutz, M. H., and Rohde, L. A. (2008). The -1021 C/T DBH polymorphism is associated with neuropsychological performance among children and adolescents with ADHD. *Am. J. Med. Genet. Part B, Neuropsychiatr. Genet. Off. Publ. Int. Soc. Psychiatr. Genet.* 147B, 485–490. doi:10.1002/ajmg.b.30636.

Kim, J. I., Kim, J.-W., Lee, J.-M., Yun, H. J., Sohn, C.-H., Shin, M.-S., et al. (2018). Interaction between DRD2 and lead exposure on the cortical thickness of the frontal lobe in youth with attention-deficit/hyperactivity disorder. *Prog. Neuropsychopharmacol. Biol. Psychiatry* 82, 169–176. doi:10.1016/j.pnpbp.2017.11.018.

Konofal, E., Lecendreux, M., Arnulf, I., and Mouren, M.-C. (2004). Iron deficiency in children with attention-deficit/hyperactivity disorder. *Arch. Pediatr. Adolesc. Med.* 158, 1113–1115. doi:10.1001/archpedi.158.12.1113.

Kwon, H. J., Ha, M., Jin, H. J., Hyun, J. K., Shim, S. H., Paik, K. C., et al. (2015). Association between BDNF gene polymorphisms and attention deficit hyperactivity disorder in Korean children. *Genet. Test. Mol. Biomarkers* 19, 366–371. doi:10.1089/gtmb.2015.0029.

Lahat, E., Heyman, E., Livne, A., Goldman, M., Berkovitch, M., and Zachor, D. (2011). Iron deficiency in children with attention deficit hyperactivity disorder. *Isr. Med. Assoc. J.* 13, 530–533.

Lee, W.-S., Lim, Y.-H., Kim, B.-N., Shin, C. H., Lee, Y. A., Kim, J. I., et al. (2020). Residential pyrethroid insecticide use, urinary 3-phenoxybenzoic acid levels, and attention-deficit/hyperactivity disorder-like symptoms in preschool-age children: The Environment and Development of Children study. *Environ. Res.* 188, 109739. doi:10.1016/j.envres.2020.109739.

Levitan, R. D., Masellis, M., Basile, V. S., Lam, R. W., Jain, U., Kaplan, A. S., et al. (2002). Polymorphism of the serotonin-2A receptor gene (HTR2A) associated with childhood attention deficit hyperactivity disorder (ADHD) in adult women with seasonal affective disorder. *J. Affect. Disord.* 71, 229–233. doi:10.1016/s0165-0327(01)00372-x.

Luca, P., Laurin, N., Misener, V. L., Wigg, K. G., Anderson, B., Cate-Carter, T., et al. (2007). Association of the dopamine receptor D1 gene, DRD1, with inattention symptoms in families selected for reading problems. *Mol. Psychiatry* 12, 776–785. doi:10.1038/sj.mp.4001972.

Mahmoud, M. M., El-Mazary, A.-A. M., Maher, R. M., and Saber, M. M. (2011). Zinc, ferritin, magnesium and copper in a group of Egyptian children with attention deficit hyperactivity disorder. *Ital. J. Pediatr.* 37, 60. doi:10.1186/1824-7288-37-60.

Mariggiò, M. A., Palumbi, R., Vinella, A., Laterza, R., Petruzzelli, M. G., Peschechera, A., et al. (2021). DRD1 and DRD2 Receptor Polymorphisms: Genetic Neuromodulation of the Dopaminergic System as a Risk Factor for ASD, ADHD and ASD/ADHD Overlap. *Front. Neurosci.* 15, 705890. doi:10.3389/fnins.2021.705890.

Mill, J., Fisher, N., Curran, S., Richards, S., Taylor, E., and Asherson, P. (2003). Polymorphisms in the dopamine D4 receptor gene and attention-deficit hyperactivity disorder. *Neuroreport* 14, 1463–1466. doi:10.1097/00001756-200308060-00011.

Németh, N., Kovács-Nagy, R., Székely, A., Sasvári-Székely, M., and Rónai, Z. (2013). Association of impulsivity and polymorphic microRNA-641 target sites in the SNAP-25 gene. *PLoS One* 8, e84207. doi:10.1371/journal.pone.0084207.

Neville, M. J., Johnstone, E. C., and Walton, R. T. (2004). Identification and characterization of ANKK1: a novel kinase gene closely linked to DRD2 on chromosome band 11q23.1. *Hum. Mutat.* 23, 540–545. doi:10.1002/humu.20039.

Nyman, E. S., Ogdie, M. N., Loukola, A., Varilo, T., Taanila, A., Hurtig, T., et al. (2007). ADHD candidate gene study in a population-based birth cohort: association with DBH and DRD2. *J. Am. Acad. Child Adolesc. Psychiatry* 46, 1614–1621. doi:10.1097/chi.0b013e3181579682.

Ouellet-Morin, I., Wigg, K. G., Feng, Y., Dionne, G., Robaey, P., Brendgen, M., et al. (2008). Association of the dopamine transporter gene and ADHD symptoms in a Canadian population-based sample of same-age twins. *Am. J. Med. Genet. Part B, Neuropsychiatr. Genet. Off. Publ. Int. Soc. Psychiatr. Genet.* 147B, 1442–1449. doi:10.1002/ajmg.b.30677.

Pan, Y.-Q., Qiao, L., Xue, X.-D., and Fu, J.-H. (2015). Association between ANKK1 (rs1800497) polymorphism of DRD2 gene and attention deficit hyperactivity disorder: a meta-analysis. *Neurosci. Lett.* 590, 101–105. doi:10.1016/j.neulet.2015.01.076.

Park, S., Jung, S.-W., Kim, B.-N., Cho, S.-C., Shin, M.-S., Kim, J.-W., et al. (2013a). Association between the GRM7 rs3792452 polymorphism and attention deficit hyperacitiveity disorder in a Korean sample. *Behav. Brain Funct.* 9, 1. doi:10.1186/1744-9081-9-1.

Park, S., Kim, B.-N., Cho, S.-C., Kim, J.-W., Kim, J. I., Shin, M.-S., et al. (2014). The metabotropic glutamate receptor subtype 7 rs3792452 polymorphism is associated with the response to methylphenidate in children with attention-deficit/hyperactivity disorder. *J. Child Adolesc. Psychopharmacol.* 24, 223–227. doi:10.1089/cap.2013.0079.

Park, T. W., Park, Y. H., Kwon, H. J., and Lim, M. H. (2013b). Association between TPH2 gene polymorphisms and attention deficit hyperactivity disorder in Korean children. *Genet. Test. Mol. Biomarkers* 17, 301–306. doi:10.1089/gtmb.2012.0376.

Pinto, R., Asherson, P., Ilott, N., Cheung, C. H. M., and Kuntsi, J. (2016). Testing for the mediating role of endophenotypes using molecular genetic data in a twin study of ADHD traits. *Am. J. Med. Genet. Part B, Neuropsychiatr. Genet. Off. Publ. Int. Soc. Psychiatr. Genet.* 171, 982–992. doi:10.1002/ajmg.b.32463.

Pliszka, S. R., Maas, J. W., Javors, M. A., Rogeness, G. A., and Baker, J. (1994). Urinary catecholamines in attention-deficit hyperactivity disorder with and without comorbid anxiety. *J. Am. Acad. Child Adolesc. Psychiatry* 33, 1165–1173. doi:10.1097/00004583-199410000-00012.

Puentes-Rozo, P. J., Acosta-López, J. E., Cervantes-Henríquez, M. L., Martínez-Banfi, M. L., Mejia-Segura, E., Sánchez-Rojas, M., et al. (2019). Genetic Variation Underpinning ADHD Risk in a Caribbean Community. *Cells* 8. doi:10.3390/cells8080907.

Ribasés, M., Ramos-Quiroga, J. A., Hervás, A., Sánchez-Mora, C., Bosch, R., Bielsa, A., et al. (2012). Candidate system analysis in ADHD: evaluation of nine genes involved in dopaminergic neurotransmission identifies association with DRD1. *world J. Biol. psychiatry Off. J. World Fed. Soc. Biol. Psychiatry* 13, 281–292. doi:10.3109/15622975.2011.584905.

Sánchez-Mora, C., Ramos-Quiroga, J.-A., Garcia-Martínez, I., Fernàndez-Castillo, N., Bosch, R., Richarte, V., et al. (2013). Evaluation of single nucleotide polymorphisms in the miR-183-96-182 cluster in adulthood attention-deficit and hyperactivity disorder (ADHD) and substance use disorders (SUDs). *Eur. Neuropsychopharmacol. J. Eur. Coll. Neuropsychopharmacol.* 23, 1463–1473. doi:10.1016/j.euroneuro.2013.07.002.

Šerý, O., Paclt, I., Drtílková, I., Theiner, P., Kopečková, M., Zvolský, P., et al. (2015). A 40-bp VNTR polymorphism in the 3’-untranslated region of DAT1/SLC6A3 is associated with ADHD but not with alcoholism. *Behav. Brain Funct.* 11, 21. doi:10.1186/s12993-015-0066-8.

Shahin, O., Meguid, N. A., Raafat, O., Dawood, R. M., Doss, M., Bader El Din, N. G., et al. (2015). Polymorphism in variable number of tandem repeats of dopamine d4 gene is a genetic risk factor in attention deficit hyperactive egyptian children: pilot study. *Biomark. Insights* 10, 33–38. doi:10.4137/BMI.S18519.

Shang, C.-Y., Gau, S. S.-F., Liu, C.-M., and Hwu, H.-G. (2011). Association between the dopamine transporter gene and the inattentive subtype of attention deficit hyperactivity disorder in Taiwan. *Prog. Neuropsychopharmacol. Biol. Psychiatry* 35, 421–428. doi:10.1016/j.pnpbp.2010.08.016.

Shim, S.-H., Hwangbo, Y., Kwon, Y.-J., Jeong, H.-Y., Lee, B.-H., Hwang, J.-A., et al. (2010). A case-control association study of serotonin 1A receptor gene and tryptophan hydroxylase 2 gene in attention deficit hyperactivity disorder. *Prog. Neuropsychopharmacol. Biol. Psychiatry* 34, 974–979. doi:10.1016/j.pnpbp.2010.05.006.

Tang, Y., Buxbaum, S. G., Waldman, I., Anderson, G. M., Zabetian, C. P., Köhnke, M. D., et al. (2006). A single nucleotide polymorphism at DBH, possibly associated with attention-deficit/hyperactivity disorder, associates with lower plasma dopamine beta-hydroxylase activity and is in linkage disequilibrium with two putative functional single nucleotide polymorphisms. *Biol. Psychiatry* 60, 1034–1038. doi:10.1016/j.biopsych.2006.02.017.

Valbonesi, S., Magri, C., Traversa, M., Faraone, S. V, Cattaneo, A., Milanesi, E., et al. (2015). Copy number variants in attention-deficit hyperactive disorder: identification of the 15q13 deletion and its functional role. *Psychiatr. Genet.* 25, 59–70. doi:10.1097/YPG.0000000000000056.

Walitza, S., Renner, T. J., Dempfle, A., Konrad, K., Wewetzer, C., Halbach, A., et al. (2005). Transmission disequilibrium of polymorphic variants in the tryptophan hydroxylase-2 gene in attention-deficit/hyperactivity disorder. *Mol. Psychiatry* 10, 1126–1132. doi:10.1038/sj.mp.4001734.

Wang, Y., Wang, T., Du, Y., Hu, D., Zhang, Y., Li, H., et al. (2021). Polygenic risk of genes involved in the catecholamine and serotonin pathways for ADHD in children. *Neurosci. Lett.* 760, 136086. doi:10.1016/j.neulet.2021.136086.

Wu, L. H., Cheng, W., Yu, M., He, B. M., Sun, H., Chen, Q., et al. (2017). Nr3C1-Bhlhb2 Axis Dysregulation Is Involved in the Development of Attention Deficit Hyperactivity. *Mol. Neurobiol.* 54, 1196–1212. doi:10.1007/s12035-015-9679-z.

Wu, L. H., Peng, M., Yu, M., Zhao, Q. L., Li, C., Jin, Y. T., et al. (2015). Circulating MicroRNA Let-7d in Attention-Deficit/Hyperactivity Disorder. *Neuromolecular Med.* 17, 137–146. doi:10.1007/s12017-015-8345-y.

Xu, Q., Ou, J., Zhang, Q., Tang, R., Wang, J., Hong, Q., et al. (2019). Effects of Aberrant miR-384-5p Expression on Learning and Memory in a Rat Model of Attention Deficit Hyperactivity Disorder. *Front. Neurol.* 10, 1414. doi:10.3389/fneur.2019.01414.

Yu, C.-J., Du, J.-C., Chiou, H.-C., Chung, M.-Y., Yang, W., Chen, Y.-S., et al. (2016). Increased risk of attention-deficit/hyperactivity disorder associated with exposure to organophosphate pesticide in Taiwanese children. *Andrology* 4, 695–705. doi:10.1111/andr.12183.

Zhang, Q., Chen, X., Li, S., Yao, T., and Wu, J. (2021). Association between the group III metabotropic glutamate receptor gene polymorphisms and attention-deficit/hyperactivity disorder and functional exploration of risk loci. *J. Psychiatr. Res.* 132, 65–71. doi:10.1016/j.jpsychires.2020.09.035.
